# Characterization of Genetic Diversity and Genome-Wide Association Mapping of Three Agronomic Traits in Qingke Barley (*Hordeum Vulgare* L.) in the Qinghai-Tibet Plateau

**DOI:** 10.3389/fgene.2020.00638

**Published:** 2020-07-03

**Authors:** Zhiyong Li, Namgyal Lhundrup, Ganggang Guo, Kar Dol, Panpan Chen, Liyun Gao, Wangmo Chemi, Jing Zhang, Jiankang Wang, Tashi Nyema, Dondrup Dawa, Huihui Li

**Affiliations:** ^1^Institute of Crop Sciences, Chinese Academy of Agricultural Sciences, Beijing, China; ^2^State Key Laboratory of Hulless Barley and Yak Germplasm Resources and Genetic Improvement, Tibet Academy of Agriculture and Animal Sciences, Lhasa, China; ^3^Tibet Agricultural and Animal Husbandry College, Nyingchi, China; ^4^International Maize and Wheat Improvement Center (CIMMYT), Texcoco, Mexico

**Keywords:** qingke barley, genetic diversity, GWAS, EigenGWAS, adaptation

## Abstract

Barley (*Hordeum vulgare* L.) is one of the most important cereal crops worldwide. In the Qinghai-Tibet Plateau, six-rowed hulless (or naked) barley, called “qingke” in Chinese or “nas” in Tibetan, is produced mainly in Tibet. The complexity of the environment in the Qinghai-Tibet Plateau has provided unique opportunities for research on the breeding and adaptability of qingke barley. However, the genetic architecture of many important agronomic traits for qingke barley remains elusive. Heading date (HD), plant height (PH), and spike length (SL) are three prominent agronomic traits in barley. Here, we used genome-wide association (GWAS) mapping and GWAS with eigenvector decomposition (EigenGWAS) to detect quantitative trait loci (QTL) and selective signatures for HD, PH, and SL in a collection of 308 qingke barley accessions. The accessions were genotyped using a newly-developed, proprietary genotyping-by-sequencing (tGBS) technology, that yielded 14,970 high quality single nucleotide polymorphisms (SNPs). We found that the number of SNPs was higher in the varieties than in the landraces, which suggested that Tibetan varieties and varieties in the Tibetan area may have originated from different landraces in different areas. We have identified 62 QTLs associated with three important traits, and the observed phenotypic variation is well-explained by the identified QTLs. We mapped 114 known genes that include, but are not limited to, vernalization, and photoperiod genes. We found that 83.87% of the identified QTLs are located in the non-coding regulatory regions of annotated barley genes. Forty-eight of the QTLs are first reported here, 28 QTLs have pleotropic effects, and three QTL are located in the regions of the well-characterized genes *HvVRN1, HvVRN3*, and *PpD-H2*. EigenGWAS analysis revealed that multiple heading-date-related loci bear signatures of selection. Our results confirm that the barley panel used in this study is highly diverse, and showed a great promise for identifying the genetic basis of adaptive traits. This study should increase our understanding of complex traits in qingke barley, and should facilitate genome-assisted breeding for qingke barley improvement.

## Introduction

Barley (*Hordeum vulgare* L.) was domesticated in Israel and Jordan in the southern part of the Fertile Crescent approximately 10,000 years ago (Badr et al., 2000). With an average world production of 120 Mt annually (Ullrich, 2010), barley ranks fourth among the most important cereal crops in the world (http://faostat.fao.org). Barley is mainly used for food, fodder, alcoholic beverage ingredient, and is generally considered to be a healthful food (Blake et al., 2010; Collins et al., 2010). In Qinghai-Tibet Plateau, six-rowed hulless (or naked) barley, called “qingke” in Chinese or “nas” in Tibetan, is mainly produced in Tibet, and Qinghai, Sichuan, and Yunnan provinces of China. In the Qinghai-Tibet plateau, Tibetans use qingke barley to make wine and for consumption (Tashi et al., 2013). As the main food of Tibetans, qingke barley has been grown on the Qinghai-Tibet Plateau for at least 3,500 years, most probably following its introduction via northern Pakistan, India and Nepal (Zeng et al., 2018). Tibetans have a rich spiritual and cultural connection to qingke barley on the Qinghai-Tibet Plateau due to its wide range of medicinal and nutritional uses. Therefore, analysis of the genetic diversity present in cultivated varieties of qingke barley is especially important.

The adaptation to diverse, high elevation environments makes qingke barley a unique resource for genetic study and barley breeding (Zeng et al., 2015). At present, the genetic architecture of grain starch quality (Li et al., 2014) and drought stress tolerance (Zeng et al., 2016) has been studied in qingke barley, and salt and aluminum tolerance have been studied in Tibetan wild barley (Qiu et al., 2011; Wu et al., 2011; Cai et al., 2013). In other studies, diverse barley lines from different regions, including the US (Zhou and Steffenson, 2013; Genievskaya et al., 2018), Europe (Xu et al., 2018), and India (Visioni et al., 2018), were used to identify the genetic architecture of complex traits (heading time, number of kernels per spike, grain yield) and disease resistance (durable spot, stripe rust) in barley. Although some studies used worldwide collections of barley germplasm, few have included barley varieties from Tibet (Pasam et al., 2012; Gyawali et al., 2017). Over the past decade, studies in barley (Cuesta-Marcos et al., 2008), wheat (Kiseleva et al., 2016), and rice (Yan et al., 2011) have shown that variations in heading date (HD), plant height (PH), and spike length (SL) contribute to environmental adaptation in cereal crops and also influence grain yield. In earlier studies, bi-parental mapping populations were used to reliably detect QTL for HD, PH, and spike morphological traits (Lin et al., 1998; Sameri et al., 2006; Zhang et al., 2009). With the emergence of more cost-effective, high-throughput genotyping technologies, single nucleotide polymorphisms (SNPs) related to HD have been identified by genome-wide association studies (GWAS) (Pasam et al., 2012; Visioni et al., 2013; Genievskaya et al., 2018), PH (Alqudah et al., 2016; Almerekova et al., 2019), leaf area (Alqudah et al., 2018), spike architecture (Comadran et al., 2011) and grain yield (Ingvordsen et al., 2015; Xu et al., 2018) in barley. However, the genetic study of complex agronomic traits in qingke barley is limited (Zhang et al., 2019).

For HD, important genes have been successfully isolated and characterized in barley. Exposure to low temperatures is known as vernalization, which is related to annual differences in seed production and flowering. This process protects the flowering meristem, which is sensitive to the cold, during winter (Yan et al., 2003; Trevaskis et al., 2006). Three genes control the vernalization parameters and growth conditions of barley: *HvVRN1, HvVRN2*, and *HvVRN3*. These are found on the respective chromosome arms 5HL, 4HL, and 7HS, all of which have been isolated (Laurie et al., 1995; Yan et al., 2003, 2004, 2006). A MADS-box transcription factor (TF) is encoded by *HvVRN1*, which shares homology with APETALA1, CAULIFLOWER, and FRUITFULL. These are transcription factors that promote flowering in the apical meristem of *Arabidopsis* (Trevaskis et al., 2003; Yan et al., 2003; Trevaskis, 2010). A transcription factor with a zinc finger-CCT domain is encoded by *HvVRN2*. While *Arabidopsis* has no homologous gene, its function is similar to *FLOWERING LOCUS C* (*FLC*), which inhibits flowering (Yan et al., 2004). *FLOWERING LOCUS T* (*FT*) in *Arabidopsis* is similar to *HvVRN3* in that it induces the expression of *HvVRN1* during periods of long daylight, promoting flowering (Yan et al., 2006; Distelfeld et al., 2009). In barley and wheat, *HvVRN3* integrates the photoperiod and vernalization pathways (Distelfeld et al., 2009). Another important pathway is that of the photoperiod, which regulates the date of flowering and heading and uses plant response daylight and optical cues from light receptors. It has been shown that *Ppd-H1* is the ortholog of the wheat *Ppd-D1* gene, a member of the pseudoresponse regulator (*PRR*) gene family via homology-based cloning (Beales et al., 2007). The major determinants of the long-day response in barley are the *Photoperiod-H1* (*Ppd-H1*) and *Photoperiod-H2* (*Ppd-H2*) genes on chromosomes 2H and 1H, respectively (Abdullaev et al., 2017). The results of the study of Turner et al. (2005) suggest that *Ppd-H1* might affect flowering by altering the expression of photoperiod pathway genes that are under circadian control. The dominant allele of *Ppd-H1* regulates response to increased photoperiod length and premature earing during long days. The recessive allele *ppd-H1* induces delays in heading during long days, while *Ppd-H2*, a dominant allele, quickens heading during short days. The recessive allele impedes it.

For PH, semi-dwarf genes include *uzu1, ari-e*, and *sdw1* genes are widely used in modern barley improvement (Kuczynska et al., 2013; Dockter and Hansson, 2015). The *ari-e* gene has served in European cultivars and been located on chromosome 5HL (Froster, 2001). The *uzu* gene, the primary dwarfing gene of East Asian barley strains, is located on chromosome 3HL (Zhang, 2000; Chono et al., 2003). Dwarfism regulated by *uzu* is induced by the mutation of one nucleotide interchange in the *HvBRI1* gene, which involves brassinolide in the response (Chono et al., 2003). The chromosome 3HL is also the site of the *sdw1* gene, which is an important dwarfing gene in Europe, North America, South America, and Australia breeding programs (Jia et al., 2009; Xu et al., 2017). The dwarfism controlled by *sdw1* caused by a deletion mutation in the gibberellin 20-oxidase gene (*HvGA20ox2*) (Xu et al., 2017). Previous studies have shown that the QTLs controlling PH and SL are distributed on multiple chromosomes (Gyenis et al., 2007; Pasam et al., 2012; Fakheri et al., 2018), and that QTLs for PH and SL are identified on different chromosomes in different environments and treatments (Gyenis et al., 2007; Fakheri et al., 2018). In a wild x cultivated barley cross, Gyenis et al. (2007) identified QTLs for PH on chromosomes 1H, 2H, 3H, and 7H, and for SL on chromosomes 1H, 2H, 3H, and 6H. Another study identified QTLs for PH on chromosomes 2H, 3H, 4H, 5H, 6H, and 7H in a spring barley collection (Pasam et al., 2012). A recent study suggests that QTLs for PH are distributed on chromosomes 5H and 7H and for SL on chromosomes 1H, 2H, 5H, and 6H in Western European barley cultivars exposed to drought (Fakheri et al., 2018).

As the growth range of barley increased, it adapted to a wide spectrum of agricultural conditions. Studying selection signals in the barley genome is important to help us understand how this genome reacted to the various agricultural conditions experienced during domestication (Russell et al., 2016). Zeng et al. (2015) resequenced the genomes of 10 Tibetan wild barley accessions to uncover patterns of adaptation to the stressful environment of the Tibetan plateau. Further resequencing of 177 Tibetan barley genomes was performed to better understand the selection markers for the adaptation of local highland barley in the exome capture target range of the genome using the fixation index (*F*_ST_) approach (Zeng et al., 2018). Eight regions as possible selective regions were identified, including the location near the *Naked caryopsis* (*nud*) on chromosome 7H. Recently, EigenGWAS, which combines the statistical framework of GWAS with eigenvector decomposition, is a novel approach for identifying regions of the genome under selection in any genetic data where the underlying population structure is unknown. EigenGWAS has been applied to studies in evolution, ecology, breeding, and human genetics (https://github.com/gc5k/GEAR/wiki/EigenGWAS).

In the present study, we collected old local qingke barley landraces in Tibet, modern qingke barley varieties, and representative qingke barley varieties from regions surrounding the Tibetan region. The genetic diversity was compared between the landraces and the two variety groups, and the trends in the changes in genetic structure from the landraces to the breeding varieties was considered. In this study, therefore, our objectives were to use the 14,970 high quality SNPs discovered using genotyping-by-sequencing (tGBS) in 308 qingke barley accessions to (1) understand the genetic diversity in the landraces and the modern varieties and the changes in population structure that occurred going from the landraces to the breeding varieties, (2) identify genetic loci associated with HD, PH, and SL by GWAS, and (3) identify loci that underwent selection for environmental adaptation using EigenGWAS. The findings of this study could facilitate a better understanding of the genetic mechanisms underlying the establishment of adaptive traits and genome-assisted selection in qingke barley breeding.

## Materials and Methods

### Plant Materials

A total of 308 qingke barley accessions were used in this study; 206 qingke landraces, 72 qingke varieties, and 30 varieties (including 18, 5, 1, and 6 varieties from Qinghai, Gansu, Yunnan, and Sichuan provinces of China, respectively). All the 308 accessions were planted in Tibet at three locations; Lhasa (N29°36′, E91°06′) in April 2018, Namling (N29°18′, E88°46′) on May 2017, and Nyingchi (N29°39′, E94°21′) in October 2017 with three replicates each. We used a randomized design to construct the field experiment. At each location, 30 seeds of each accession were planted in a plot with two rows of 150 cm long and 30 cm between rows. HD was measured as the number of days when the head first emerged from the flag leaf sheath on the main shoot in a plot (Zadoks scale, *Z* = 50; Hemming et al., 2009). The PH was measured as the above-ground plant height without the awns. The SL was measured as the length from the base of main spike to the tip of main spike (excluding awns). All traits were measured as the average of five random plants.

### Phenotypic Data Analysis

The Pearson's correlation coefficients between the traits and the broad sense heritability (*H*^2^) of target traits were calculated by AOV functionality in QTL IciMapping v.4.1 (Meng et al., 2015). In the analysis of variance of the three traits, variance components were estimated from a linear model; phenotype was partitioned into overall mean, genotypic effect, replication effect (i.e., location), and random error effect, all of which were treated as fixed effects. The *H*^2^ on plot level was estimated from the following equation:

$$\begin{array}{l} H^{2}=\frac{\sigma_{G}^{2}}{\sigma_{G}^{2}+\sigma_{\varepsilon }^{2}}, \end{array}$$

where $\sigma_{G}^{2}$ is the genetic variance and $\sigma_{\varepsilon }^{2}$ is the variance of the error. Although 308 accessions were planted at three locations, HD, PH, and SL were not all measured in three locations. Only SL has high-quality data in two locations (Nyingchi and Namling) in Tibet, abbreviated as SL_NC and SL_NM, respectively. For HD ad PH, phenotype from one location was used, HD in Lhasa (abbreviated as HD_LS) and PH in Namling (abbreviated as PH_NM), since from other two locations either the *H*^2^ were lower than 30%, or only one measurement was available for each plant. Considering data with low heritability was not reliable to conduct GWAS, and data with no replication could not be used to estimate the *H*^2^ and evaluate the data quality, we discarded the low-quality data. For clarity, HD_LS, PH_NM, SL_NC, and SL_NM were used in the following-up analysis.

### SNP Genotyping and Genotypic Data Analyses

The 308 accessions were genotyped using a newly developed genotyping-by-sequencing technology (tGBS) that eases the process of sorting high-quality GBS sequencing libraries and results in more accurate SNP calling (Ott et al., 2017; Li et al., 2019). Sequence reads were aligned to the *Hordeum vulgare* Hv IBSC PGSB v2 reference genome (Mascher et al., 2017) after de-barcoding and trimming. SNP calling was conducted using only those reads that aligned to a single location in the reference genome. In total, 46,034 polymorphic sites for each accession were discovered, and the data was filtered as follows: missing values ≤ 0.4, heterozygosity rate (Het. Rate) ≤ 0.2, and minor allele frequency (MAF) ≥0.05 (Supplementary Table 1). After filtering, 14,970 high-quality SNPs were retained in the follow-up analysis. To assess population diversity, genome-wide pairwise linkage disequilibrium (LD) was calculated between SNP pairs to investigate the potential of the array to capture all significant regions associated with the observed phenotypes using the software package TASSEL v5.2 (Bradbury et al., 2007). LD was estimated by using the squared allele-frequency correlation (*r*^2^; Weir and Cockerham, 1996) for pairs of loci, since *r*^2^ is affected not only by recombination frequencies at the two sites, but also by the differences in allele frequencies between sites. Decay of LD was evaluated, as was the distance between sites in base pairs (bp) with non-linear regression as implemented in the R package (Remington et al., 2001). To avoid multiple significances within individual LD blocks, the support interval was determined when the decay distance of LD reached *r*^2^= 0.5. Nucleotide diversity (π) across the barley genome was calculated with TASSEL v5.2. The population structure of the 308 accessions was evaluated using principle component analysis (PCA) and a phylogenetic tree. Pairwise distances were estimated between genotyped individuals using an unbiased model of substitution frequencies. Distance estimates were then used to construct a phylogenetic tree using the Neighbor-Joining-like algorithm described by Saitou and Nei (1987) and implemented in the NJS module of the APE R package (Paradis et al., 2004). Unlike conventional neighbor-joining methods, the NJS algorithm is tolerant of missing data, enabling its use with GBS data. Relative branch lengths are proportional to the amount of divergence observed between individuals. The effective sample size was calculated according to the method in Powell et al. (2010) as implemented in the software GEAR.

### GWAS Analysis

A GWAS for the three agronomic traits was conducted with a general linear model (GLM) and a mixed linear model (MLM) as implemented in TASSEL v5.2 software (Bradbury et al., 2007). For both models, the first principal component of the PCA was fitted as the cofactor to exclude the effect of population structure. In MLM, a variance–covariance kinship matrix, as covariates to estimate the association between phenotypes and genotypes (Zhang et al., 2010), was also considered. To declare QTL from the GWAS results, the phenotypic observation of SL was reshuffled 1,000 times to analyze the null distribution. We calculated the 95th quantile of the 1,000 most significant *p*-values over 1,000 permutations to be 5.18 after *log*_10_ transformation. The Bonferroni correction, –*log*_10_(1/14,970) = 4.18, was also calculated. To balance the false positives and false negatives, a –*log*_10_(*P*) threshold of 4.00 was used for the GLM and 3.00 was used for the MLM. To determine whether the uncovered genetic architecture was appropriate, the identified QTL was used to predict the performance of the corresponding trait. The most significant SNP in each QTL region was fitted in the linear model with the original trait performance as the dependent variable. The adjusted coefficient of determination (*R*^2^) from the linear model was then calculated. The performance of QTL in different locations were estimated by $a=\frac{1}{e}\sum_{i=1}^{e}a_{i}$ and *ae_i_* = *a_i_* - *a*, where *a* was the averaged effect of QTL across locations, *a*_*i*_ was the additive effect of QTL for each location, *e* is the number of locations, and *ae*_*i*_ was the additive by environment effect of QTL in each location (Li et al., 2015).

### Analysis of Gene Annotation and Enrichment

We used SnpEff to conduct functional annotations and effect predictions of the target SNPs (Cingolani et al., 2012). The Barley Hv_IBSC_PGSB_v2 reference genome gene annotation was downloaded as a gff3 file from the Ensembl plants database (http://plants.ensembl.org/index.html). Gene annotation information was acquired by BARLEX: The Barley Genome Explorer (https://apex.ipk-gatersleben.de/apex/f?p=284:10::::::; Colmsee et al., 2015). A Singular Enrichment Analysis (SEA) tool was used to perform a functional enrichment analysis of the annotated genes (Tian et al., 2017).

### EigenGWAS Analysis

EigenGWAS is a regression approach based on principal component analysis (Chen et al., 2016; Li et al., 2019). It is similar to GWAS; however, the phenotype is replaced with an eigenvector (EV) to capture genetic variation in the studied population. In this study, EigenGWAS, implemented in the software GEAR (https://github.com/gc5k/GEAR), was used to separate loci under selection by treating top 10 eigenvectors (i.e., EV1-EV10) as phenotypes. We adjusted the *p*-value using a genomic control factor, denoted as *P*_*GC*_, to exclude the effect of the genetic drift (Devlin and Roeder, 1999), and used the *P*_*GC*_ to identify loci under selection. We reshuffled the first eigenvector 1,000 times to identify the significance cutoff for the relevant loci, which helped us analyze the null distribution. We calculated the 95th quantile of the 1,000 most significant *p*-values over 1,000 permutations to be 5.75 after *log*_10_ transformation. Considering the Bonferroni correction 4.18 as mentioned above, a –*log*_10_(*P*) threshold of 4.00 was applied for EigenGWAS analyses in all 10 eigenvectors.

## Results

### Phenotypic Variation and Correlation Analysis

**Figure 1:**
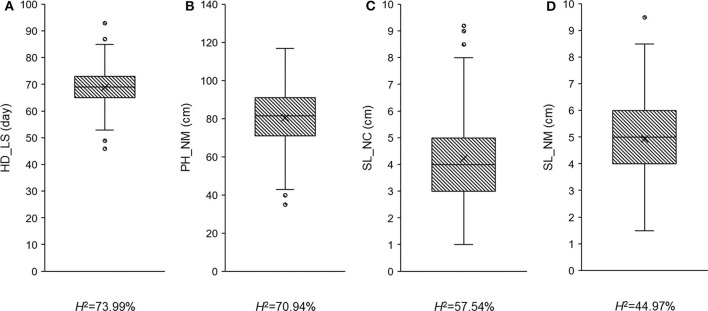
The phenotypic distribution and heritability in broad sense for heading date in Lhasa (HD_LS) **(A)**, plant height in Namling (PH_NM) **(B)**, spike length in Nyingchi (SL_NC) **(C)**, and spike length in Namling (SL_NM) **(D)**.

To determine whether the observed traits exhibit wide variation, are highly heritable, and/or display a normal distribution, the recorded phenotypic data was analyzed using ANOVA (Table 1 and Supplementary Table 2) and boxplots (Figure 1). Fifty-one plants had no measurement for SL_NC, so the degrees of freedom in this case was only 256 (Table 1). All the variance components were significant (*P* < 0.05) across trials, with the exception of the replicates in PH_NM and SL_NM (Table 1). Wide variations ranging from 46 to 93 days in HD_LS, from 35 to 117 cm in PH_NM, from 1 to 9.2 cm in SL_NC, and from 1.5 to 9.5 cm in SL_NM were observed in the collection of 308 qingke barley accessions (Figure 1). The SL distribution showed that the SL_NM mean was higher than it was for SL_NC (Figures 1C,D), and the correlation between SL_NM and SL_NC was 0.21 (*P* < 0.01; Supplementary Figure 1). The reason for this may be due to the big environmental difference between Namling (4,000 m above sea level) and Nyingchi (2,995 m above sea level) and the overcast and rainy weather in Nyingchi at flowering time, which was not conducive to pollination and thus decreased the effective seed-setting rate of the barley spikes. The broad-sense heritabilities for the three observed traits ranged from 44.97 to 73.99% (Figure 1). The highest correlation was between PH_NM and SL_NM (i.e., 0.48 with *P* < 0.01; Supplementary Figure 1), and there was a negative correlation between SL_NC and HD_LS. These observations are consistent with the general experience regarding the relationships between PH and SL (Wang et al., 2010), and between SL_NC and HD_LS (Wang et al., 2010; Al-Tabbal and Al-Fraihat, 2012).

**Table 1:** Analysis of variance (ANOVA) of three traits across three locations.

**Trait**	**Source**	**DF^e^**	**Sum of square**	**Mean square**	***F*-Value**	**Pr > *F***
HD_LS^a^	Genotype	307	22587.70	73.58	9.44	0.00
	Replicate	2	5627.66	2813.83	361.03	0.00
PH_NM^b^	Genotype	307	142700.59	464.82	8.32	0.00
	Replicate	2	27.28	13.64	0.24	0.78
SL_NC^c^	Genotype	256	1016.82	3.97	5.04	0.00
	Replicate	2	6.21	3.11	3.95	0.02
SL_NM^d^	Genotype	307	1010.40	3.29	3.45	0.00
	Replicate	2	5.26	2.63	2.76	0.06

a *HD_LS, heading date in Lhasa*.

b *PH_NM, plant height in Namling*.

c *SL_NC, spike length in Nyingchi*.

d *SL_NM, spike length in Namling*.

e *DF, degree of freedom*.

### Genetic Diversity and Population Structure in the 308 Qingke Barley Accessions

**Figure 2:**
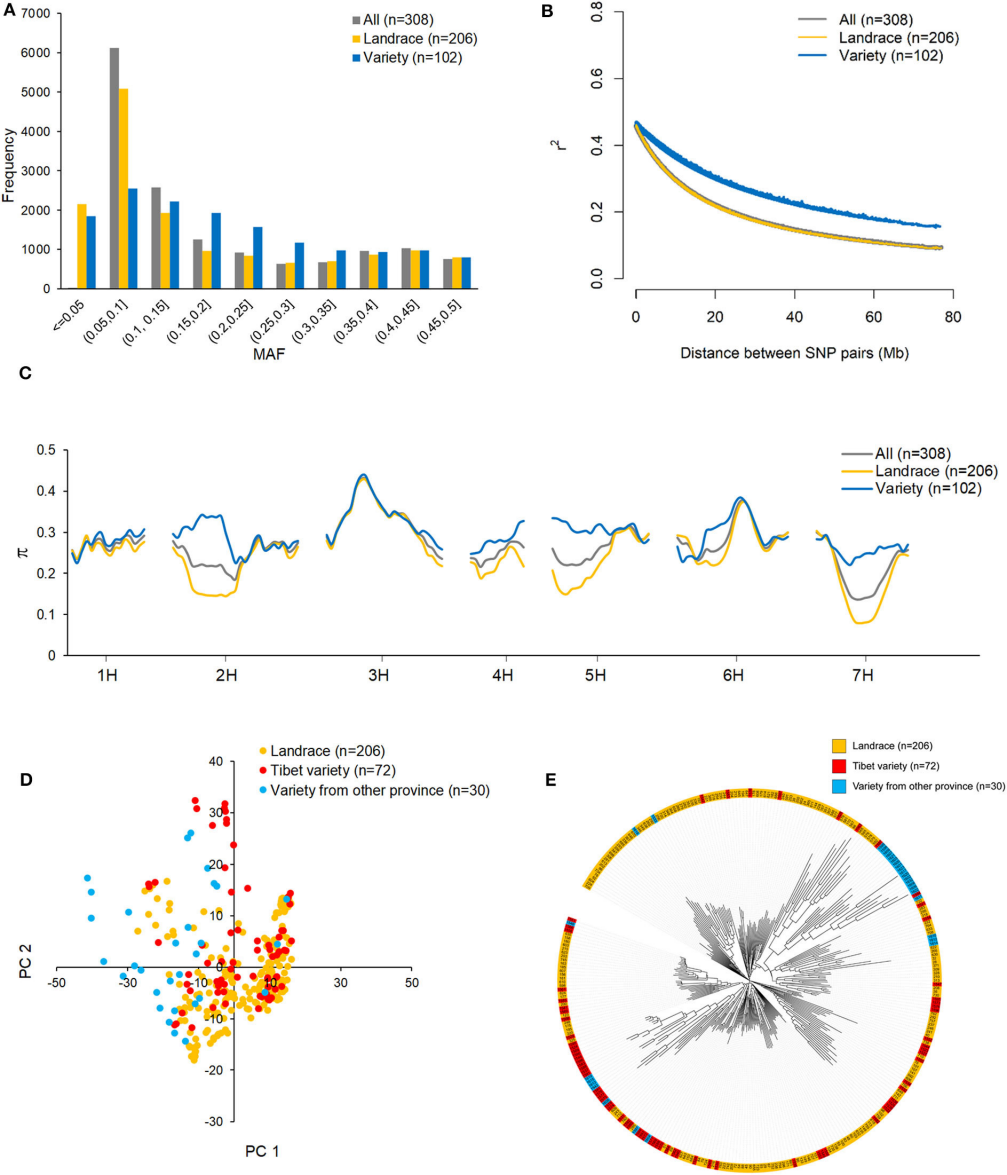
The distribution of minor allele frequency (MAF) **(A)**, linkage disequilibrium (LD) decay **(B)**, and nucleotide diversity (π) **(C)** across the barley genome in all 308 highland barley accessions, 206 landraces, and 102 varieties; the population structure of 308 barley accessions evaluated by principle component analysis (PCA) **(D)** and phylogenetic tree **(E)** base on 14970 high-quality SNPs.

The MAF distributions of all 14,970 SNPs in the whole dataset and in the landrace and variety subpopulations are shown in Figure 2A. Because the 14,970 SNPs were filtered to remove those with MAF < 0.05 in the 308 accessions, the minimum MAF here is 0.05, and the average MAF is 0.183. The MAF ranged from 0 to 0.5 in both subpopulations. SNPs with MAF < 0.05 were considered to be rare SNPs. In this sense, more rare SNPs were observed in the landrace subpopulation (2,150) than in the variety subpopulation (1,841). The numbers of SNPs with MAFs ranging from 0.05 to 0.1 were 5,086 and 2,545 in the landrace and variety subpopulations, respectively. This suggests that more low MAF SNPs are present in the landrace subpopulation than in the variety subpopulation. Non-linear models of LD decay for the 206 landraces and 102 varieties are shown in Figure 2B. In general, LD in both datasets showed an intermediate rate of decline. The predicted value of *r*^2^ declined to 0.5 within 1 Mb, which is considered to be the length of the support interval. As expected, LD decayed faster in the landrace subpopulation than in the variety subpopulation. The predicted value of *r*^2^ declined to 0.2 within 23 Mb for the landraces and within 49 Mb for the varieties. It remained >0.1 for over 80 Mb in the varieties. Due to the different allele distribution of the SNPs in the two subpopulations, nucleotide diversity (π) in the variety subpopulation was higher than in the landrace subpopulation, particularly on chromosomes 2H, 4H, 5H, and 7H (Figure 2C).

To determine whether the population structure could be discerned from the whole-genome genotyping data, PCA (Figure 2D) and a phylogenetic analysis (Figure 2E) were conducted for the 308 accessions. Based on the PCA plot, the two subpopulations, landraces and varieties, could not be clearly separated. This is likely due to a large proportion of the varieties being derived from qingke barley landraces. However, from the phylogenetic tree, it cannot be ruled out that the 15 varieties from Gansu and Qinghai provinces were not derived from the Tibetan landraces (Figure 2E).

### GWAS and EigenGWAS

**Figure 3:**
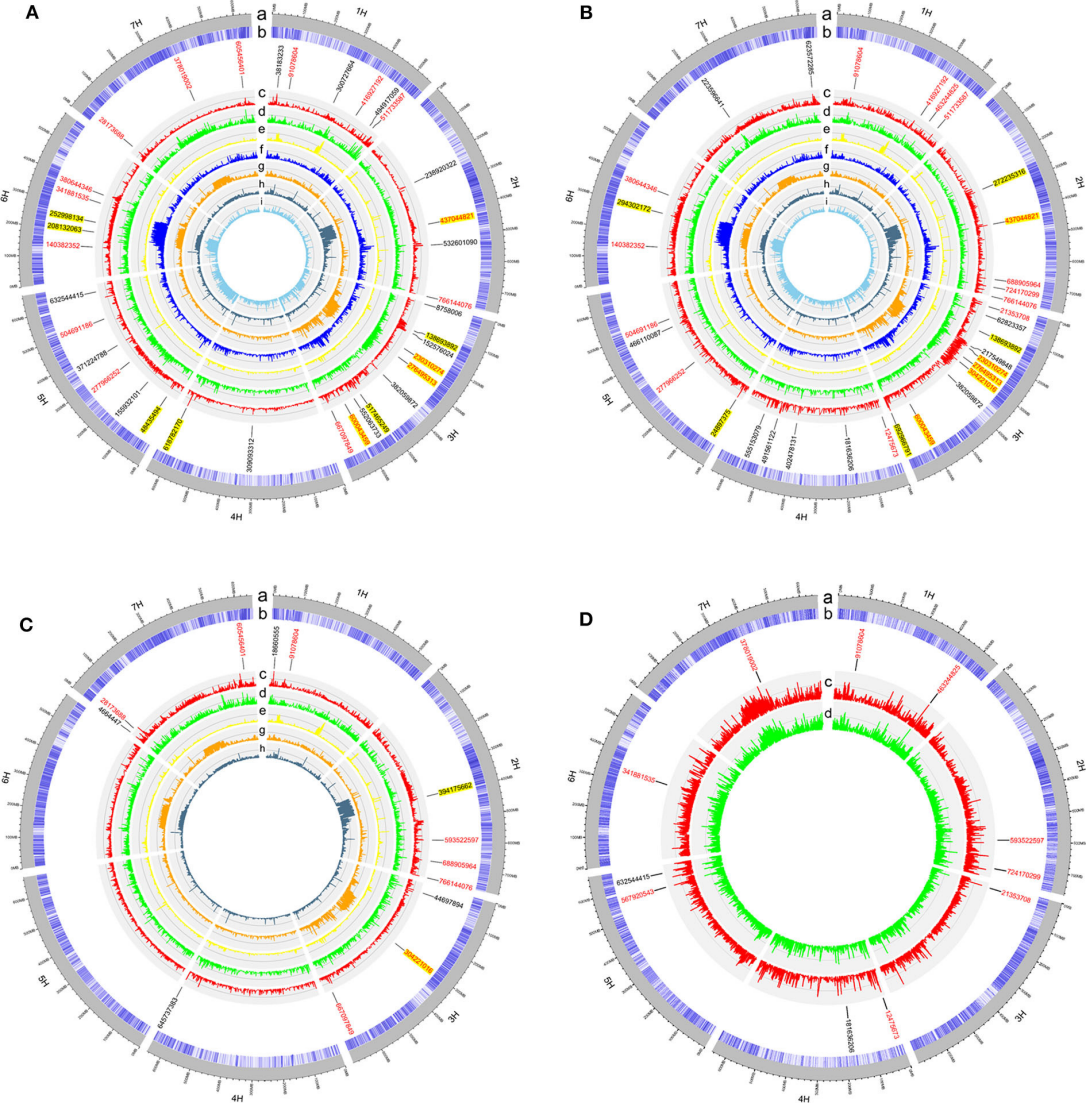
The circular plots for heading date in Lhasa (HD_LS) **(A)**, plant height in Namling (PH_NM) **(B)**, spike length in Nyingchi (SL_NC) **(C)**, and spike length in Namling (SL_NM) **(D)**. From the outer circle to the inner circle, a is for the barley genome; b is for the SNP density; c is for the manhattan plot from generalized linear model (GLM); d is for the manhattan plot from mixed linear model (MLM); e is for the manhattan plot from EigenGWAS under the tenth eigenvector (EV10); f is for the manhattan plot from EigenGWAS under the seventh eigenvector (EV7); g is for the manhattan plot from EigenGWAS under the fifth eigenvector (EV5); h is for the manhattan plot from EigenGWAS under the third eigenvector (EV3); and i is for the manhattan plot from EigenGWAS under the second eigenvector (EV2). The SNP positions associated with the trait of interest were marked in black font; of which with pleotropic effects were highlighted in red font; and detected by EigenGWAS were highlighted in yellow background.

**Figure 4:**
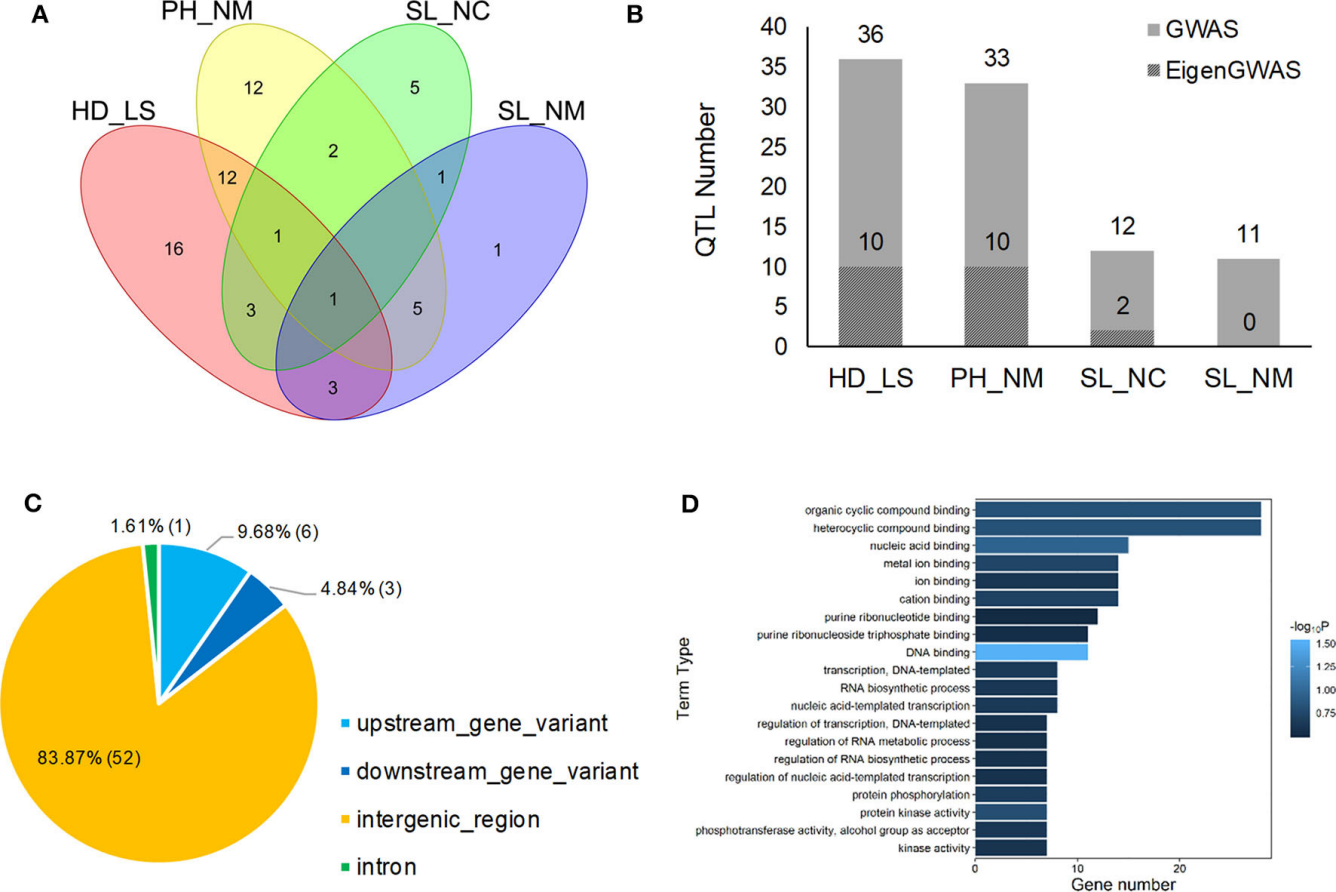
Venn plot of QTL distribution for HD_LS, PH_NM, SL_NC, and SL_NM **(A)**, the distribution of QTL number for heading date in Lhasa (HD_LS), plant height in Namling (PH_NM), spike length in Nyingchi (SL_NC), and spike length in Namling (SL_NM) **(B)**; and gene annotation **(C)** and ontology **(D)** for the 62 QTL identified by GWAS.

In the GWAS, a total of 62 QTLs distributed across the barley genome that control three agronomic traits were identified either by GLM or by MLM (Figure 3 and Table 2). To evaluate if the first PC as cofactor was appropriate, no PC and PC number with 2–5 were also used to conduct GWAS (Supplementary Figures 2–11). Results showed that the parameter estimation would be inflated if no PC as cofactor in GWAS model. The parameter estimations from PC number 1–5 were fairly the same. Of the 62 QTLs, the largest number of QTLs, 16, was distributed on chromosome 3H, and the lowest number (6) was distributed on chromosomes 6H and 7H. There were 29 QTLs (46.7%) that were detected by both GLM and MLM; 16 QTLs were declared as selection loci by EigenGWAS under five eigenvectors (i.e., EV2, EV3, EV5, EV7, and EV10); six QTLs were reported by other studies, and six QTLs were consistently identified by GLM, MLM, and EigenGWAS (Table 2). In total, 28 QTLs had pleotropic effects (red text in Figures 3, 4A). One QTL with pleotropic effects located at 91,078,604 bp on chromosome 1H was associated with all three traits in four trials. A QTL at 766,144,076 bp on chromosome 2H was related to the three traits HD_LS, PH_NM, and SL_NC, and was also associated with PH, days to seed maturation (SMT), peduncle length (PL), and HD, as reported by Genievskaya et al. (2018). Of 28 pleotropic-effect QTLs, six were detected by EigenGWAS as well, and these are shown in red text highlighted in yellow in Figures 3A–C and Table 2. These are the QTLs located at 437,044,821 bp on chromosome 2H by EV3, 138,693,892 bp on chromosome 3H by EV10, 230,310,274 bp on chromosome 3H by EV5, 276,495,313 bp on chromosome 3H by EV5, 304,221,016 bp on chromosome 3H by EV5, and 600,043,459 bp on chromosome 3H by EV10 (Figure 3 and Table 2). Two QTLs at 91,078,604 bp on chromosome 1H and at 593,522,597 bp on chromosome 2H associated with SL were both detected in two locations (Figures 3, 4A and Table 2).

**Table 2:** QTL identified by GWAS using generalized linear model (GLM) and mixed linear model (MLM) and EigenGWAS.

**Chr**.	**Pos. (bp)**	**GLM**		**MLM**		***F*st**	***–log*_**10**_(*P*_**GC**_)^a^**	**Annotation**	**References**
		***–log*_**10**_(*P*)**	**Trait**	***–log*_**10**_(*P*)**	**Trait**				
1H	18,660,555	7.81	SL_NC					upstream_gene_variant	Mikołajczak et al., 2017; Hu et al., 2018
1H	38,183,233	12.52	HD_LS					intergenic_region	
1H	91,078,604	5.36	PH_NM, SL_NC, HD_LS	3.66	SL_NM, PH_NM			intergenic_region	
1H	300,727,664	6.41	HD_LS	4.68	HD_LS			intergenic_region	
1H	416,927,192	7.08	HD_LS, PH_NM	3.47	PH_NM			intergenic_region	Genievskaya et al., 2018; Hill et al., 2019
1H	463,244,825	5.18	SL_NM	4.67	SL_NM, PH_NM			intergenic_region	
1H	494,917,059	8.18	HD_LS					upstream_gene_variant	
1H	511,733,587	8.23	HD_LS, PH_NM					intergenic_region	Alqudah et al., 2014; Almerekova et al., 2019
2H	238,920,322	8.39	HD_LS					intergenic_region	
2H	272,235,316	5.16	PH_NM			0.34	6.06 (EV3)	intergenic_region	
2H	394,175,662	4.23	SL_NC			0.30	7.34 (EV3), 5.61 (EV10)	intergenic_region	
2H	437,044,821	6.30	PH_NM, HD_LS	3.37	PH_NM	0.13	4.97 (EV3)	intergenic_region	
2H	532,601,090	10.55	HD_LS					intergenic_region	Pasam et al., 2012; Pauli et al., 2014
2H	593,522,597	4.74	SL_NC, SL_NM	3.61	SL_NM			downstream_gene_variant	Wang et al., 2016
2H	688,905,964	4.70	PH_NM, SL_NC	3.21	SL_NC			intergenic_region	
2H	724,170,299	5.47	PH_NM, SL_NM	3.08	SL_NM, PH_NM			upstream_gene_variant	Comadran et al., 2011; Pasam et al., 2012; Hu et al., 2018
2H	766,144,076	6.92	HD_LS, SL_NC, PH_NM	4.82	HD_LS			intron_variant	Genievskaya et al., 2018
3H	8,758,006	7.46	HD_LS					intergenic_region	
3H	21,353,708	5.49	PH_NM, SL_NM	3.04	SL_NM			intergenic_region	
3H	44,697,894	4.23	SL_NC	3.90	SL_NC			intergenic_region	
3H	62,823,357	4.17	PH_NM					intergenic_region	
3H	138,693,892	8.62	HD_LS	3.14	PH_NM	0.10	6.17 (EV10)	intergenic_region	
3H	152,576,024	11.42	HD_LS					intergenic_region	
3H	217,549,848	7.23	PH_NM	3.99	PH_NM			intergenic_region	
3H	230,310,274	6.67	HD_LS, PH_NM			0.57	4.48 (EV5)	intergenic_region	
3H	276,495,313	6.28	HD_LS, PH_NM			0.72	4.47 (EV5)	intergenic_region	
3H	304,221,016	7.79	PH_NM	3.39	PH_NM, SL_NC	0.71	5.25 (EV5)	intergenic_region	
3H	382,059,872	10.31	HD_LS	3.36	PH_NM			intergenic_region	
3H	517,465,249	11.80	HD_LS			0.46	4.33 (EV7)	intergenic_region	
3H	552,063,733	10.20	HD_LS					intergenic_region	
3H	600,043,459	5.30	PH_NM, HD_LS			0.08	6.73 (EV10)	intergenic_region	Tondelli et al., 2013
3H	667,097,849	10.02	HD_LS, SL_NC					intergenic_region	
3H	692,966,791	8.20	PH_NM	3.78	PH_NM	0.18	6.56 (EV2)	intergenic_region	
4H	12,475,673	4.41	SL_NM, PH_NM	3.10	SL_NM, PH_NM			upstream_gene_variant	Pauli et al., 2014
4H	181,636,206	4.43	PH_NM	3.05	SL_NM			intergenic_region	
4H	309,093,312	7.06	HD_LS					intergenic_region	
4H	402,478,131	5.10	PH_NM	3.41	PH_NM			intergenic_region	
4H	491,561,122	6.24	PH_NM	3.37	PH_NM			intergenic_region	Tondelli et al., 2013
4H	555,153,079	5.60	PH_NM					intergenic_region	
4H	618,782,170	16.60	HD_LS			0.09	6.17 (EV10)	intergenic_region	Pauli et al., 2014; Almerekova et al., 2019
4H	645,737,383	4.84	SL_NC					intergenic_region	
5H	24,897,375	8.10	PH_NM	3.16	PH_NM	0.17	4.44 (EV2)	downstream_gene_variant	
5H	48,435,494	9.26	HD_LS			0.47	4.91 (EV7)	intergenic_region	
5H	155,932,101	7.00	HD_LS	4.92	HD_LS			intergenic_region	
5H	277,966,252	6.31	HD_LS, PH_NM					intergenic_region	
5H	371,224,788	8.44	HD_LS					intergenic_region	
5H	466,110,087	5.15	PH_NM					intergenic_region	
5H	504,691,186			4.05	PH_NM, HD_LS			intergenic_region	
5H	567,920,543	4.13	SL_NM	3.41	SL_NM			intergenic_region	
5H	632,544,415	7.62	HD_LS	3.28	SL_NM			downstream_gene_variant	
6H	140,382,352	7.72	PH_NM, HD_LS					intergenic_region	
6H	208,132,063	4.43	HD_LS			0.27	4.82 (EV2), 5.21 (EV7)	intergenic_region	Genievskaya et al., 2018
6H	252,998,134	4.19	HD_LS			0.21	4.02 (EV2)	intergenic_region	
6H	294,302,172	4.97	PH_NM	4.07	PH_NM	0.33	4.88 (EV2), 4.09 (EV7)	intergenic_region	
6H	341,881,535	5.68	HD_LS	4.89	HD_LS, SL_NM			intergenic_region	
6H	380,644,346	6.30	HD_LS, PH_NM					intergenic_region	
7H	4,664,447	5.28	SL_NC					upstream_gene_variant	
7H	28,173,688	10.17	SL_NC, HD_LS					intergenic_region	
7H	223,596,641			4.86	PH_NM			intergenic_region	Pham et al., 2019
7H	378,019,002	4.09	SL_NM	4.17	HD_LS, SL_NM			intergenic_region	
7H	605,456,401	4.38	HD_LS	5.10	HD_LS, SL_NC			upstream_gene_variant	
7H	623,572,285	5.96	PH_NM	3.29	PH_NM			intergenic_region	Hu et al., 2018; Almerekova et al., 2019; Pham et al., 2019

a *Corrected p-value of EigenGWAS. Blank means the QTL was not identified by the corresponding method*.

In general, 36, 33, 12, and 11 QTLs were associated with HD_LS, PH_NM, SL_NC, and SL_NM, respectively (Figure 4B and Table 2), and are positively correlated with the broad sense heritabilities (Figure 1). In our study, we were able to investigate pleiotropy of QTLs on multiple traits. We observed that there were 16 QTLs for HD_LS and PH_NM in common. However, the correlation between HD_LS and PH_NM was not significant (Supplementary Figure 1), which may due to the repulsion linkage phase of the 16 QTLs (Supplementary Table 4). In contrast, six PH_NM QTLs were also significant for SL_NM, and the correlation between these two traits was 0.48, which was highly significant. The reason for this may be that the six QTLs are in coupling linkage phase (Supplementary Table 4). To evaluate the performance of QTL associated with SL in different locations, genotype by environment effects were estimated (Supplementary Figure 12 and Supplementary Table 5). The most significant genotype-by-environment QTLs identified by both GLM and MLM were at 593,522,597 bp on chromosome 2H and 21,353,708 bp on chromosome 3H, since their additive effects were both significant in Nyingchi, but not in Namling. In addition, there were three genotype-by-environment QTLs identified by GLM on chromosomes 1H, 2H, and 7H, respectively.

### Candidate Gene Annotation and Enrichment

The annotation conducted on the 62 significant QTLs identified by GWAS (Table 2) showed that 52 (83.87%) of QTL regions are intergenic, and that 10 (16.13%) are genic (Figure 4C). This is consistent with the *Hordeum vulgare* Hv IBSC PGSB v2 reference genome, where 19.2% of the barley genome is genic (Mascher et al., 2017) and a high ratio of loci (78.00%) related to phenotypic variation are identified in intergenic regions (Mei et al., 2017). Of the QTL, 9.68% and 4.84% were located in the upstream and downstream gene regions, and 1.61% of the QTL were in the intron regions (Figure 4C). In total, 114 known genes were mapped as significant QTLs in the GWAS, and most of them were assigned to the “molecular function” and “biological process” categories in gene ontology (GO) analysis (Figure 4D). One QTL, located at 605,456,401 bp on chromosome 7H, controls HD_LS and SL_NC, and is 2.1 Kb upstream of *HORVU7Hr1G100540*, a known gene that encodes an SBP (S-ribonuclease binding protein) family protein. A QTL at 24,897,375 on chromosome 5H significantly associated with PH_NM was found to be located 799 bp downstream of the gene *HORVU5Hr1G009980* that encodes a tetratricopeptide repeat (TPR)-like superfamily protein. For SL_NM and SL_NC, a stable QTL at 593,522,597 bp on chromosome 2H is located 554 bp downstream of *HORVU2Hr1G081800*, which encodes a WPP domain interacting protein 2 (Supplementary Table 3). In addition, screening of the associated mapping population identified variations in HD, and we found a significant SNP (5H: 599,361,872) near the vernalization gene *HvVRN1*, a significant SNP (7H: 38,508,938) near the vernalization gene *HvVRN3*, and a significant SNP (1H: 514,145,049) near the photoperiod gene *PpD-H2*.

### Phenotype Prediction

**Figure 5:**
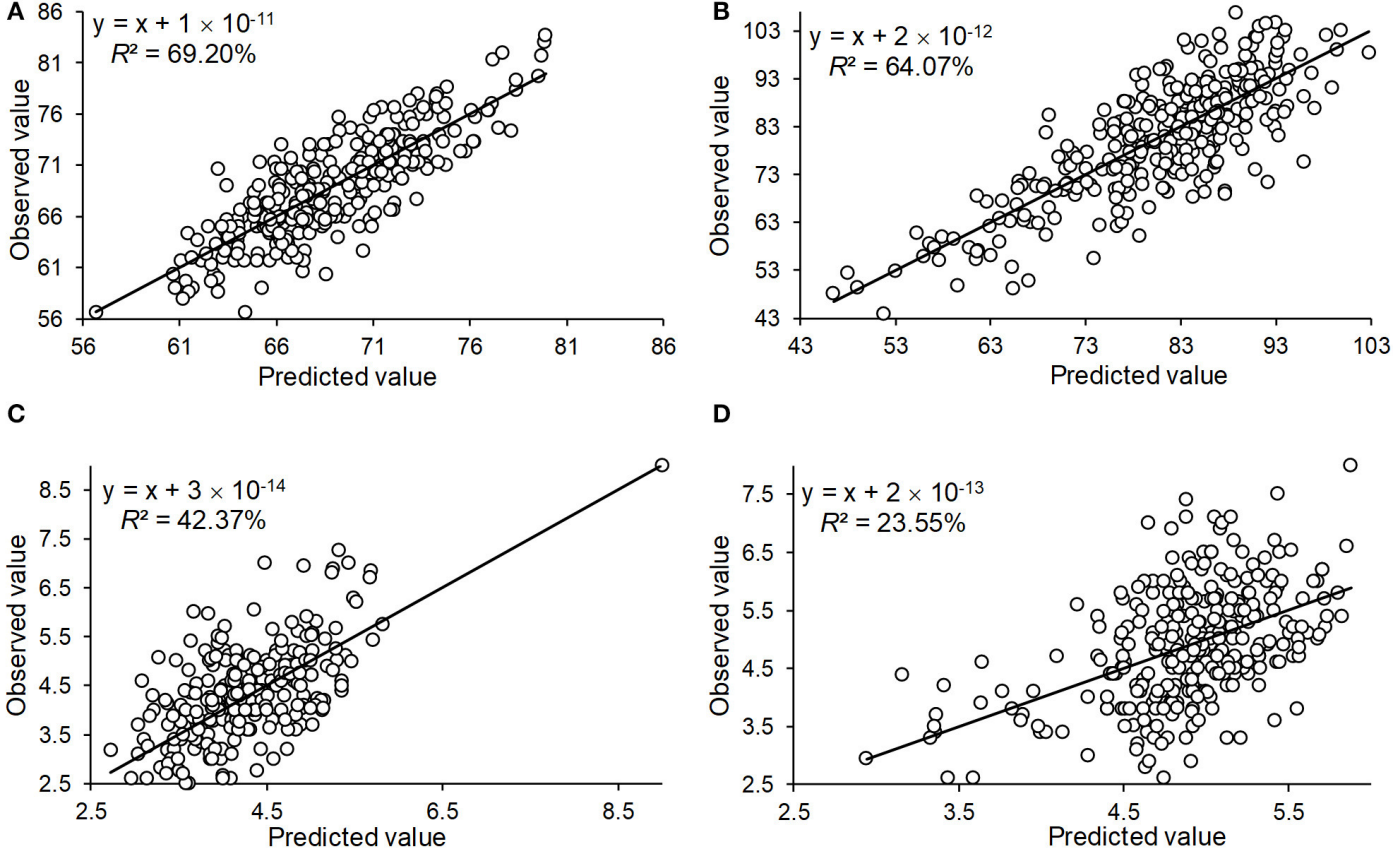
The prediction of the observed phenotype by the QTL identified by GWAS for the traits of heading date in Lhasa (HD_LS) **(A)**, plant height in Namling (PH_NM) **(B)**, spike length in Nyingchi (SL_NC) **(C)**, and spike length in Namling (SL_NM) **(D)**.

To determine the accuracy of the QTL effect estimation, we used the significant QTL additive effect estimates to predict the phenotypic observations for the three traits, and were able to accurately predict HD_LS (*R*^2^= 69.20%), PH_NM (*R*^2^= 64.07%), and SL_NC (*R*^2^= 42.37%) (Figure 5). For SL_NM, the prediction was low, due in part to the low heritability of SL in NM (Figure 1). Looking at the broad sense heritabilities for the three traits (Figure 1) suggests that the QTL results presented in this study are reliable, and provide further evidence that a large proportion of the phenotypic variation can be explained by additive variance in this association panel.

## Discussion

To the best of our knowledge, few genetic studies have investigated the complex agronomic traits in Tibetan qingke barley (Zhang et al., 2019). Previous reports have included only a limited number of qingke barley accessions to identify potential signals of adaptation and domestication. For example, 95 wild barley accessions from Tibet and 28 six-rowed hulless barley varieties from Tibet and Xinjiang were used to show that the Tibetan Plateau and the surrounding areas are primary centers of barley cultivation (Dai et al., 2012); six wild-barley genotypes collected from the Tibetan Plateau were used in an RNA-seq analysis to reveal multiple origins of barley domestication (Dai et al., 2014); 10 Tibetan wild barley accessions were re-sequenced to uncover patterns of adaptation (Zeng et al., 2015); and 177 Tibetan barley accessions were re-sequenced to identify signals of selection in the genome (Zeng et al., 2018). In contrast, 308 qingke accessions from the Qinghai-Tibet Plateau, including 278 qinke barley accessions and 30 qingke varieties collected from five other Chinese provinces, were used for this study. The effective sample size is 272 in total, which is comprised of 182.63 in the landrace subpopulation and 89.37 in the variety subpopulation. Our results demonstrate that this panel has a large effective population size with high levels of intra-species genetic flow, making it a suitable candidate for the characterization of genetic structure and adaptation, and was appropriate for the genetic study of complex traits by GWAS.

Previous studies have shown that QTLs identified on all seven chromosomes are significantly associated with HD, except for photoperiod and vernalization loci (Pasam et al., 2012). QTLs located on chromosomes 1H, 2H, and 5H have been identified that are significantly associated with PH in a worldwide spring barley investigation (Alqudah et al., 2016). Another recent study shows that QTLs on chromosomes 5H and 7H have been identified to be significantly associated with PH, and QTLs on chromosomes 1H, 2H, 5H, and 6H were shown to be significantly associated with SL in spring barley exposed to drought (Fakheri et al., 2018). In our study, SNPs that are significantly associated with HD and PH were identified on almost all barley chromosomes, and the SNPs mainly identified on chromosomes 1H, 2H, 3H, 4H, and 7H had significant effects on SL. To validate the effect estimation of each QTL, QTL effect estimations were used to predict the observed phenotypic performance. The highest prediction accuracy was 69.2% for HD, and the lowest prediction accuracy was 23.55% for SL in Namling. In the present study, heritability for all traits ranged from 44.97 and 73.99%. For these traits, both the number of detected QTL, prediction accuracy, and broad-sense heritability showed the same trend in which higher heritability corresponded to high prediction accuracy and more detected QTLs.

In order to figure out the specific QTLs for qingke barley, QTLs reported in the reference and identified in this study were aligned to the barley Hordeum vulgare Hv IBSC PGSB v2 reference genome, and their physical positions of markers were queried in BARLEX database (https://apex.ipk-gatersleben.de/apex/f?p=284:48:::NO:RP:P48_MARKER_CHOICE:4). As a result, 48 of 62 identified QTLs were first reported in this study (Table 2 and Supplementary Figure 13). For HD, 36 QTLs were identified, 7 of which were reported; for PH, 33 QTLs were identified, 9 of which were reported; and for SL, 22 QTLs were identified, 5 of which were reported. The possible reason for the high number of novel QTLs (viewed as qingke barley specific QTLs) identified in this study may be because (1) the genetics of qingke barley is lack of analysis; and (2) some reported QTLs based on SSR markers could not find the their physical positions, and some reported QTLs developed by in-house SNP chips could not match the chip version in the database. These qingke barley specific QTLs could be utilized for marker-assisted selection in qingke barley breeding programs focusing on adaption and high grain yield.

Among the five common vernalization and photoperiod loci (i.e., *HvVRN1, HvVRN2, HvVRN3, Ppd-H1*, and *Ppd-H2*), the SNPs near *HvVRN1, HvVRN3*, and *PpD-H2* were significant in this study. It suggests that *HvVRN1, HvVRN3*, and *Ppd-H2* play an important role in the qingke barley population, and should be prioritized when attempting to improve HD and plant growth in qingke barley cultivars in the qingke barley-growing regions of the Qinghai-Tibet Plateau. Previous studies have shown that QTLs for PH and SL are located on different chromosomes depending on the different environments and treatments (Gyenis et al., 2007; Fakheri et al., 2018). In the present study, we observed QTLs related to PH on all chromosomes, while QTLs associated with SL were detected on all chromosomes in Namling but on 1H, 2H, 3H, 4H, and 7H in Nyingchi. Due to the differences in locations of QTLs identified in different environments, the expression of genes controlling HD, PH, and SL are probably related to the environment and variety-specific adaptability. To validate this hypothesis, the study of selection signals in the qingke barley genome were conducted to help us understand how qingke barley how qingke barley responds to various historical environmental factors (Russell et al., 2016). Eight regions were identified as candidate selective regions, and these are distributed on all chromosomes except for chromosome 4H (Zeng et al., 2018). We used the first 10 eigenvectors for EigenGWAS and identified several selected loci in the qingke barley genome. We further compared the selected loci with the located QTLs, and found that some of these loci were located in the regions (1 Mb) of these QTLs (Table 1). Previous studies have shown that genes for HD and PH always influence barley maturity and adaptation (Barua et al., 1993; Laurie et al., 1994). In the present study, the results of EigenGWAS analysis indicated that the QTLs associated with HD and PH also bear signatures of genetic selection in this qingke barley population.

## Conclusion

In this study, we identified several genetic loci associated with SL, PH, and HD in qingke barley from the Qinghai-Tibet Plateau using 14,970 SNPs in a tGBS genotyping assay. We found that more rare SNPs (2,150) were found in the landrace subpopulation than in the variety subpopulation. That is to say, the number of SNPs was higher in the varieties than in the landraces, indicating that the varieties grown in Tibet and the varieties from around the Tibetan area may be derived from the different landraces grown in the different regions. A GWAS identified 62 QTLs that are associated with HD, PH, and SL, and 114 known genes were mapped which include, but are not limited to, genes involved in vernalization and photoperiod. Of the 62 QTLs, 48 are first reported here as qingke specific QTLs, 52 (83.87%) were found to be in intergenic regions, 28 had pleotropic effects, and three QTL were in the regions of the well-characterized genes *HvVRN1, HvVRN3*, and *PpD-H2*. In addition, by comparing signatures of selection identified by EigenGWAS and novel QTLs, we found that six QTLs related to HD and PH in qingke barley cultivars from the Qinghai-Tibet Plateau were also under selection. The findings presented here could help increase our understanding of the genetic mechanisms underlying the establishment of adaptive traits, and also enable marker-assisted selection for important traits in qingke barley breeding.

## Data Availability Statement

The datasets generated for this study can be found in the https://www.ncbi.nlm.nih.gov/sra/PRJNA606408.

## Author Contributions

This study was designed by HL and DD. The experiment was performed under the support of GG, JZ, and TN. The evaluation of traits was conducted by NL, KD, PC, LG, and WC. Data were analyzed by ZL, NL, and HL. The manuscript was drafted by ZL, NL, and HL, and revised by GG and JW. All authors contributed to the article and approved the submitted version.

## Conflict of Interest

The authors declare that the research was conducted in the absence of any commercial or financial relationships that could be construed as a potential conflict of interest.
