# Supplementary material for: Characterization of Genetic Diversity and Genome-Wide Association Mapping of Three Agronomic Traits in Qingke Barley (Hordeum Vulgare L.) in the Qinghai-Tibet Plateau
Source: Front Genet. 2020 Jul 3;11:638. doi: 10.3389/fgene.2020.00638 (PMC7351530; doi:10.3389/fgene.2020.00638)
Supplement: Supplementary file 2 [file Data_Sheet_2.docx]

Supplementary Material

# Supplementary Figures


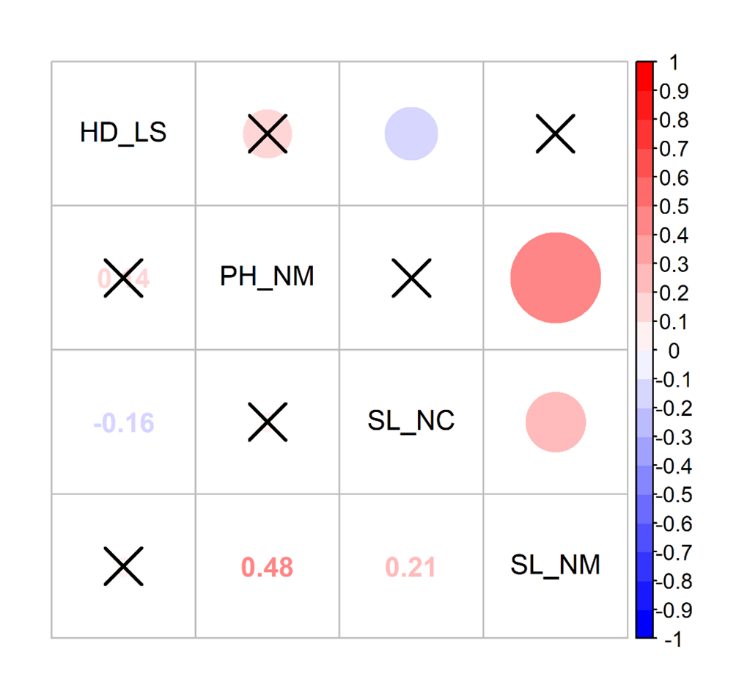


**Supplementary Figure S1.** The Pearson’s correlation coefficients between traits. The coefficient which was not reach the significance level (*P* < 0.01) were marked by cross.


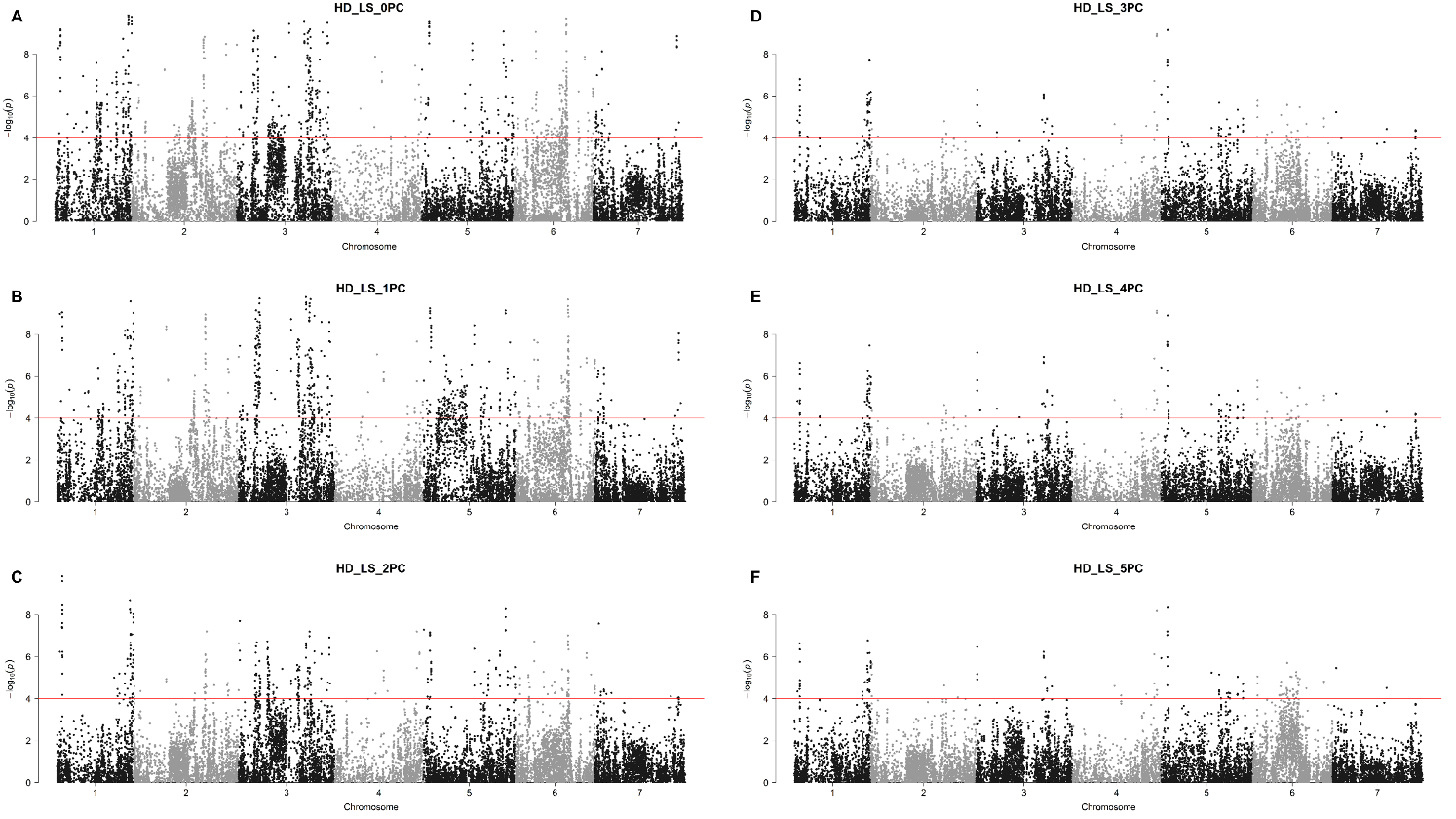


**Supplementary Figure S2.** Manhattan plots of GWAS for heading date in Lhasa (HD_LS). A to F are the results from GLM with no PC and PC number from 1 to 5 as cofactor, respectively.


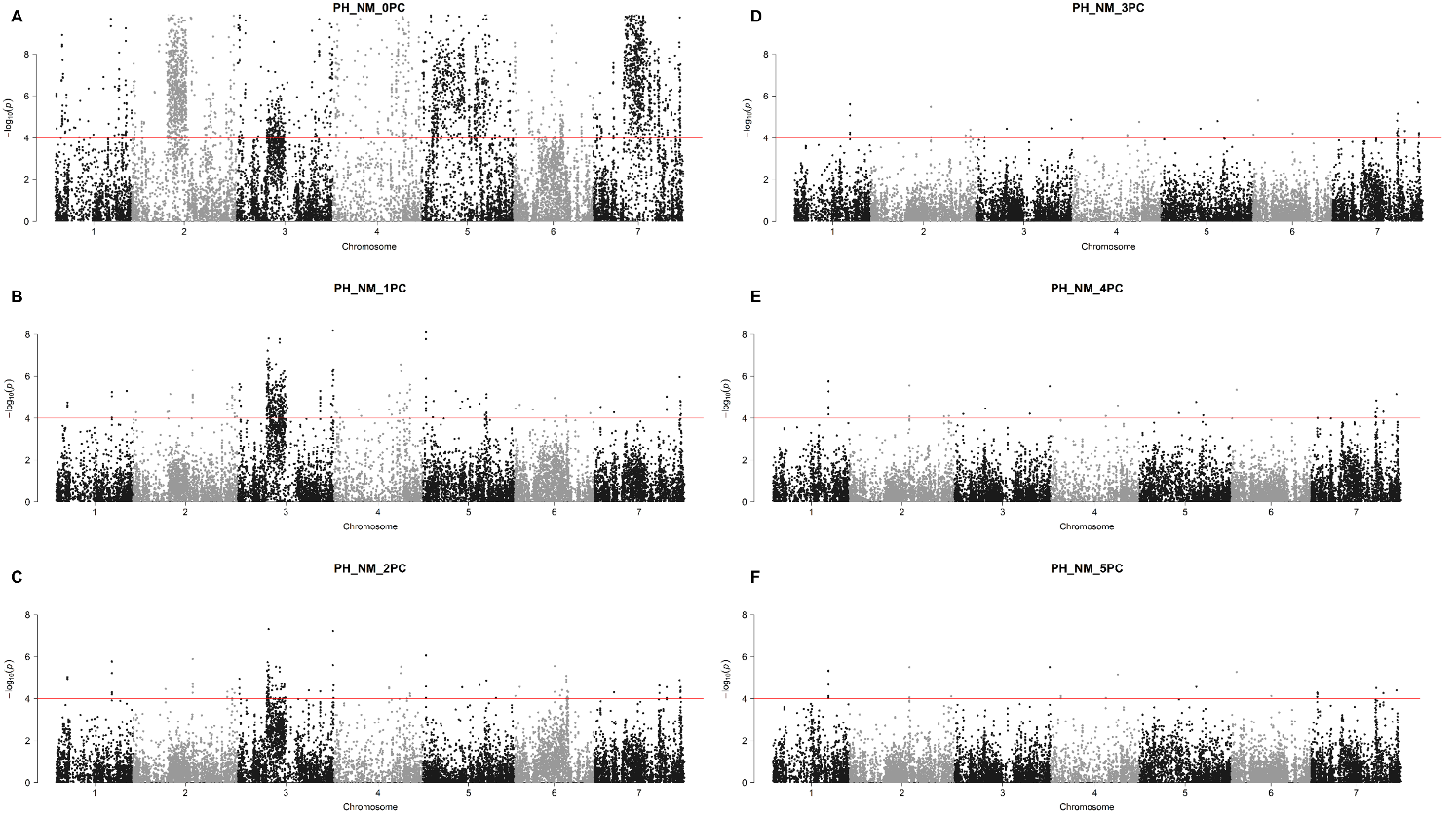


**Supplementary Figure S3.** Manhattan plots of GWAS for plant height in Namling (PH_NM). A to F are the results from GLM with no PC and PC number from 1 to 5 as cofactor, respectively.


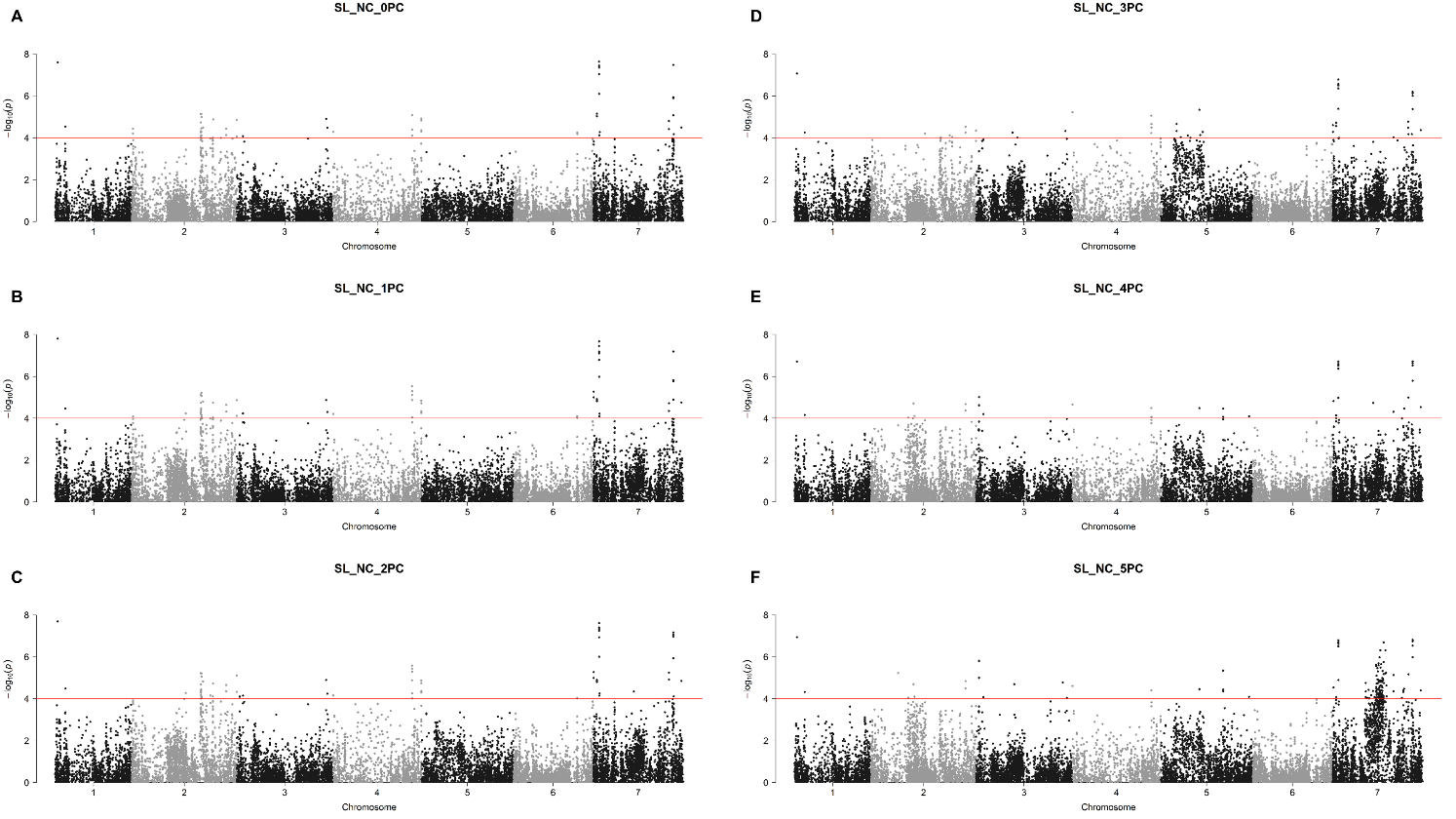


**Supplementary Figure S4.** Manhattan plots of GWAS for spike length in Nyingchi (SL_NC). A to F are the results from GLM with no PC and PC number from 1 to 5 as cofactor, respectively.


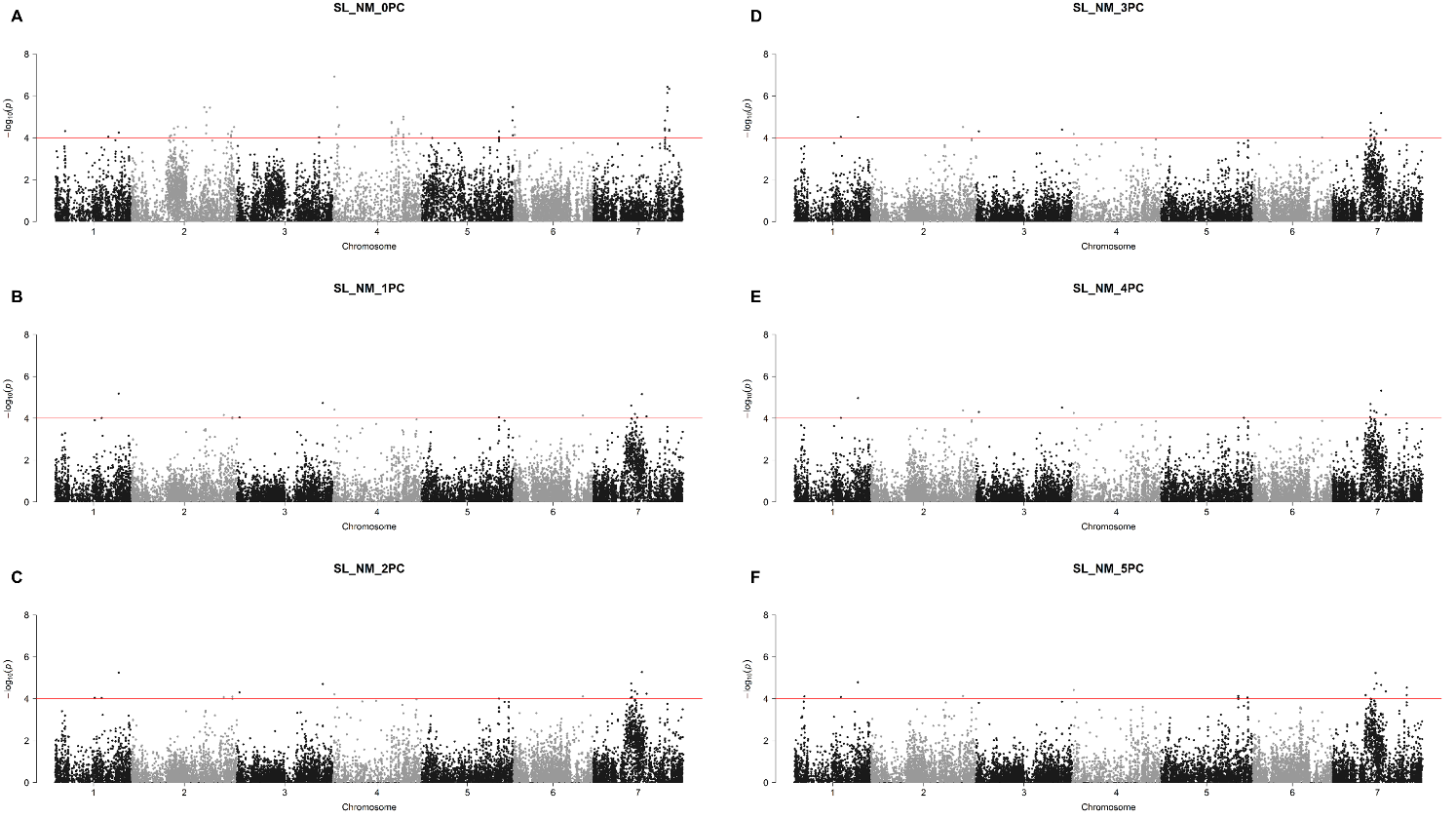


**Supplementary Figure S5.** Manhattan plots of GWAS for spike length in Namling (SL_NM). A to F are the results from GLM with no PC and PC number from 1 to 5 as cofactor, respectively.


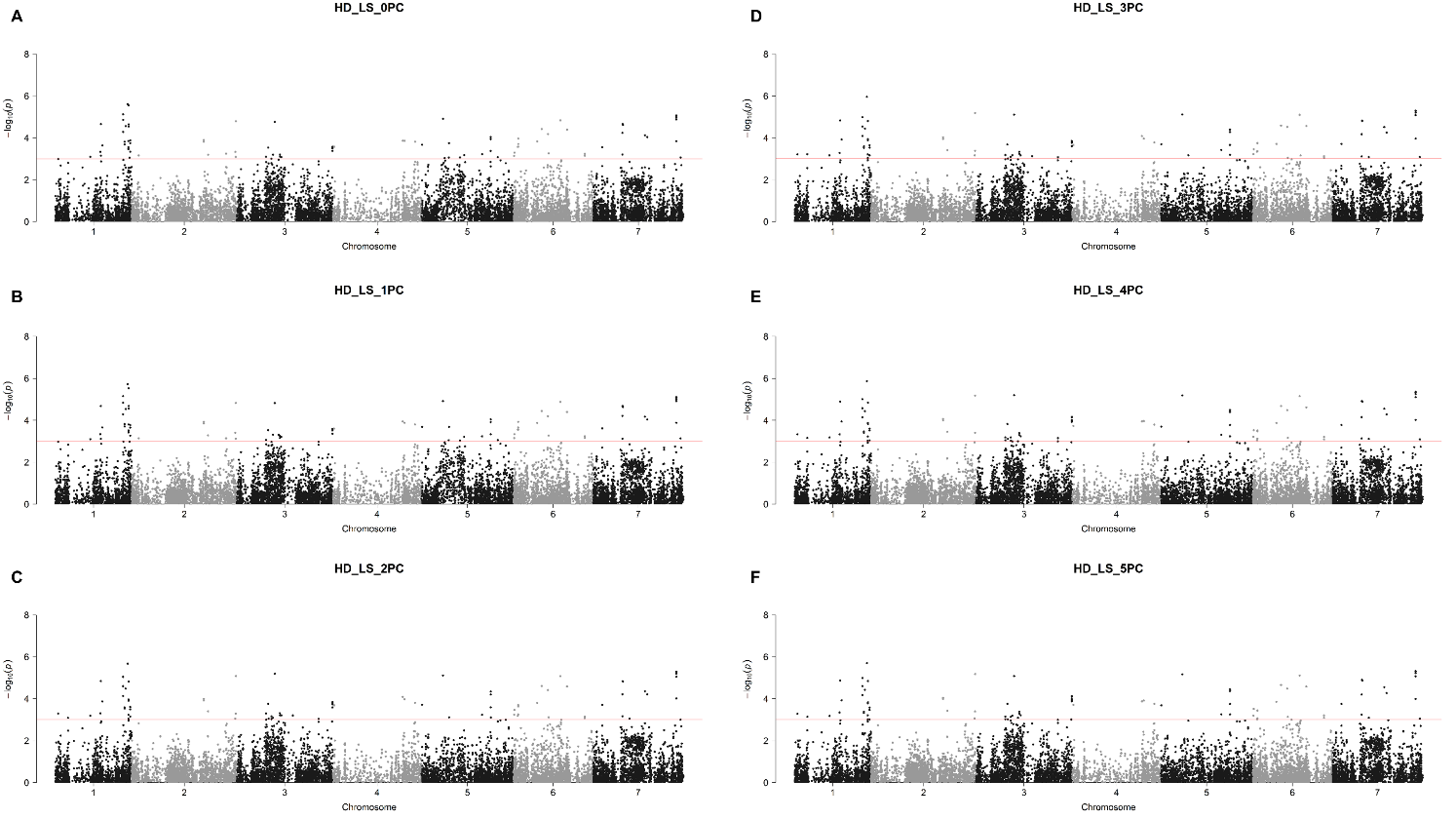


**Supplementary Figure S6.** Manhattan plots of GWAS for heading date in Lhasa (HD_LS). A to F are the results from MLM with no PC and PC number from 1 to 5 as cofactor, respectively.


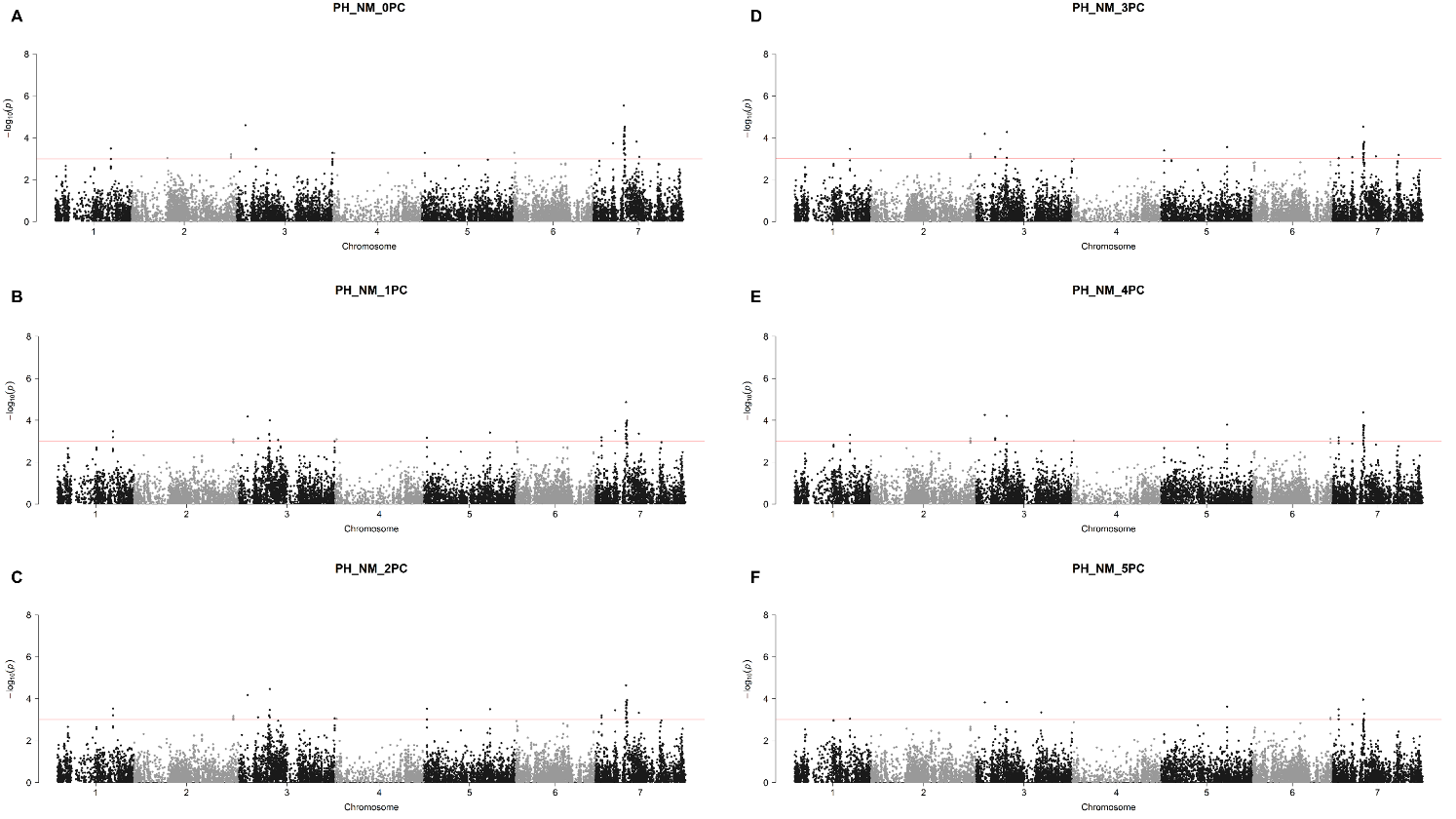


**Supplementary Figure S7.** Manhattan plots of GWAS for plant height in Namling (PH_NM). A to F are the results from MLM with no PC and PC number from 1 to 5 as cofactor, respectively.


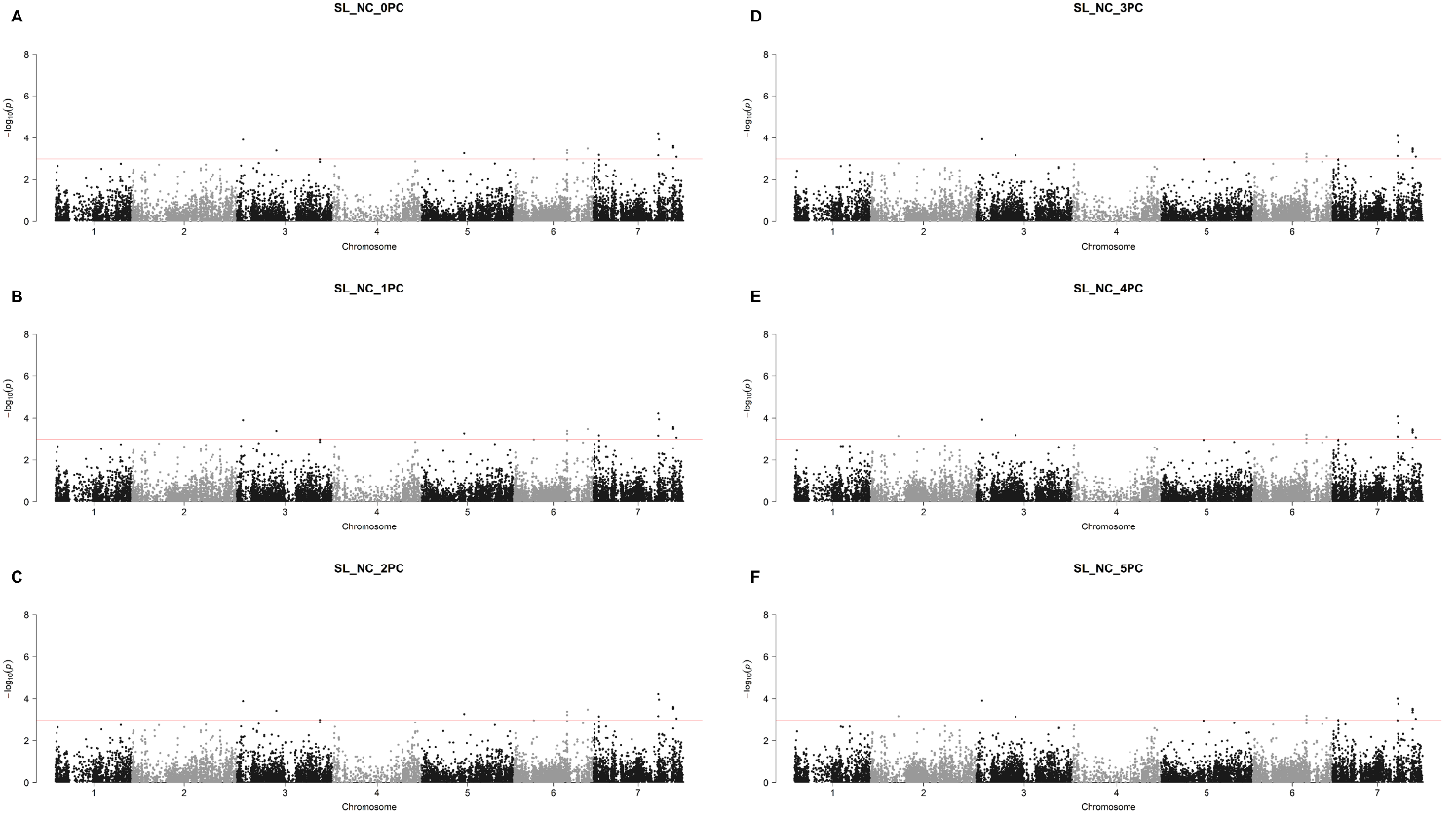


**Supplementary Figure S8.** Manhattan plots of GWAS for spike length in Nyingchi (SL_NC). A to F are the results from MLM with no PC and PC number from 1 to 5 as cofactor, respectively.


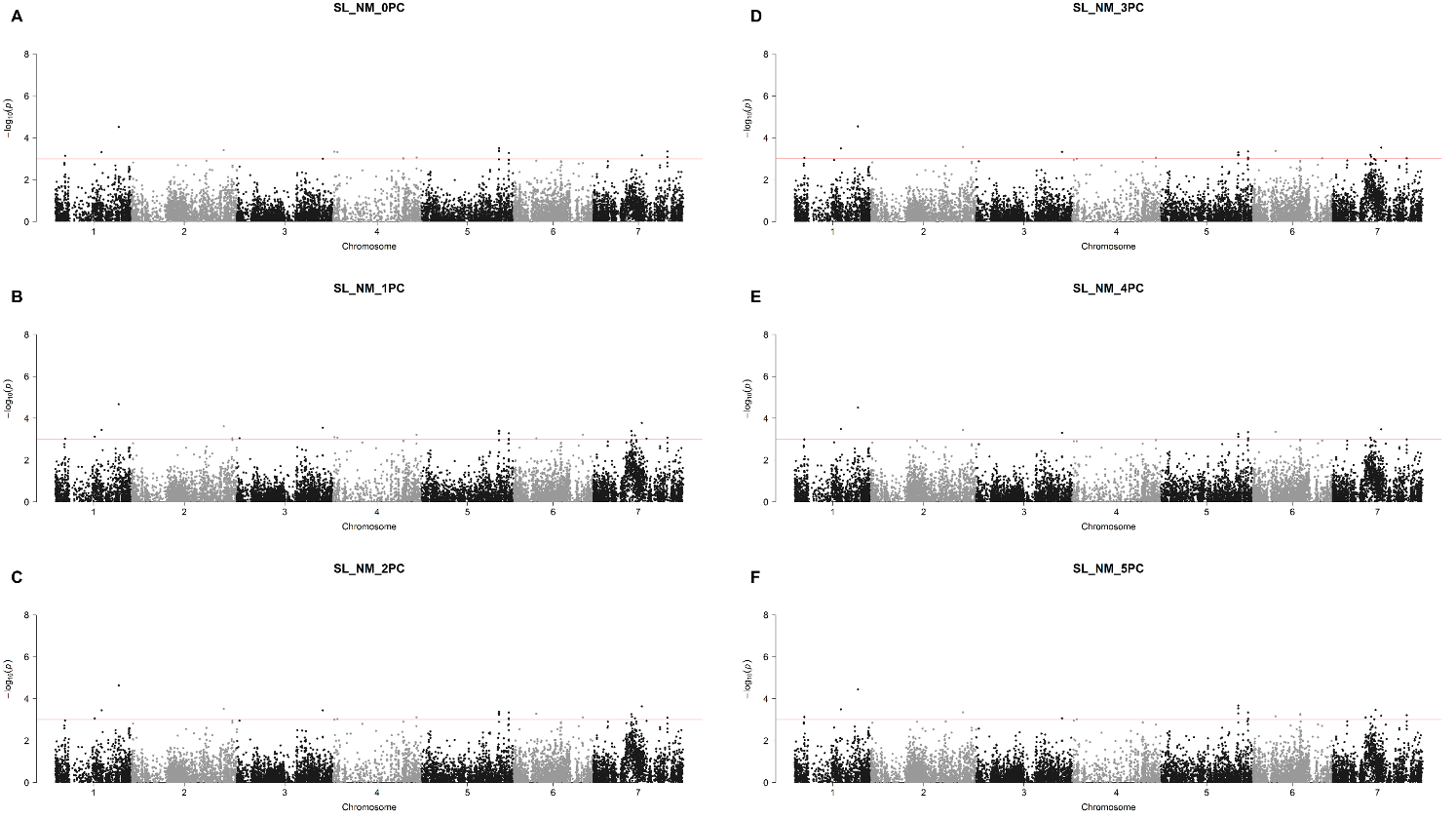


**Supplementary Figure S9.** Manhattan plots of GWAS for spike length in Namling (SL_NM). A to F are the results from MLM with no PC and PC number from 1 to 5 as cofactor, respectively.


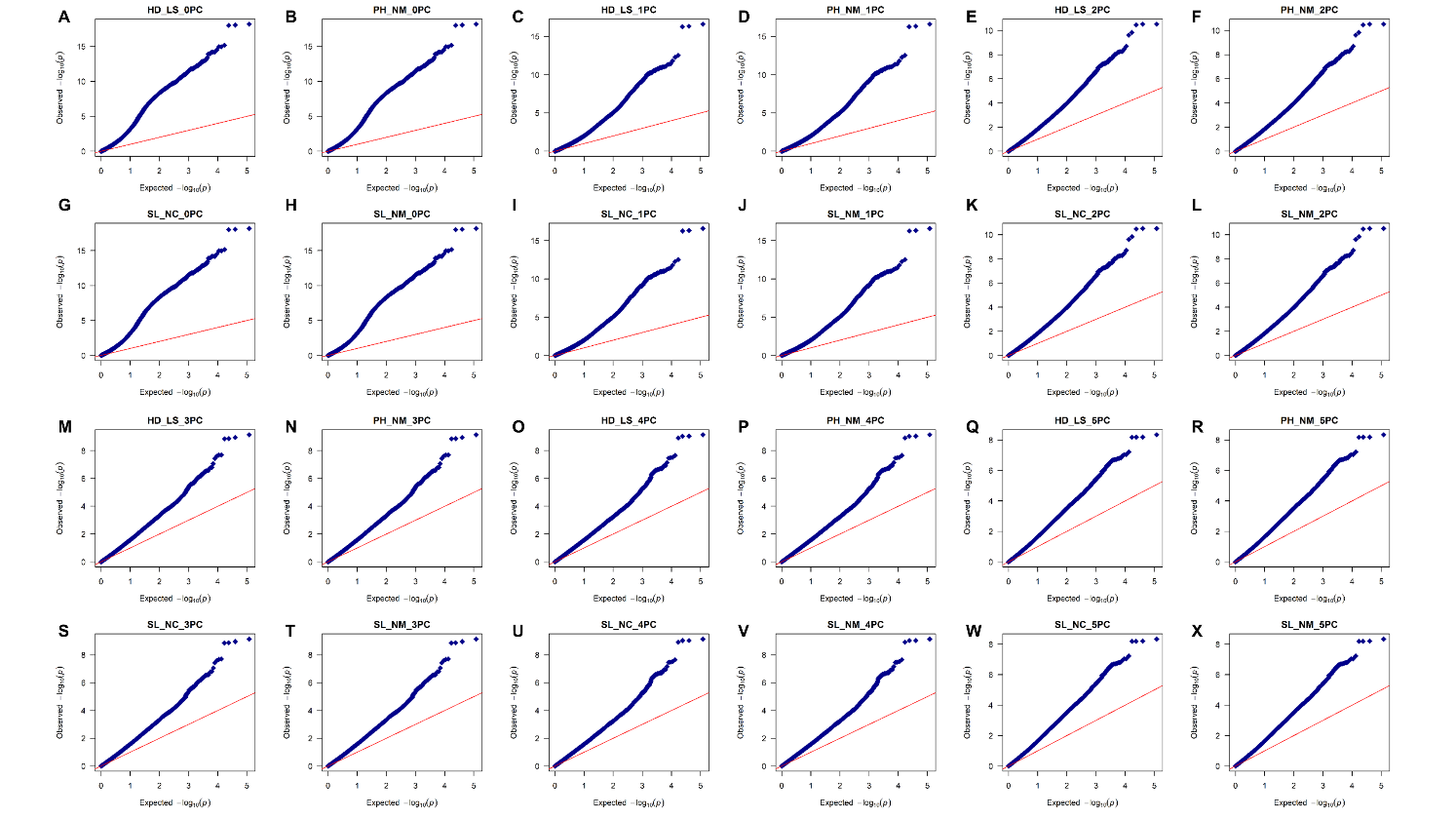


**Supplementary Figure S10.** Quantile-Quantile plots (Q-Q plot) from GWAS. A to X are for heading date in Lhasa (HD_LS), plant height in Namling (PH_NM), spike length in Nyingchi (SL_NC), and spike length in Namling (SL_NM) from GLM with no PC and PC number from 1 to 5 as cofactor, respectively.


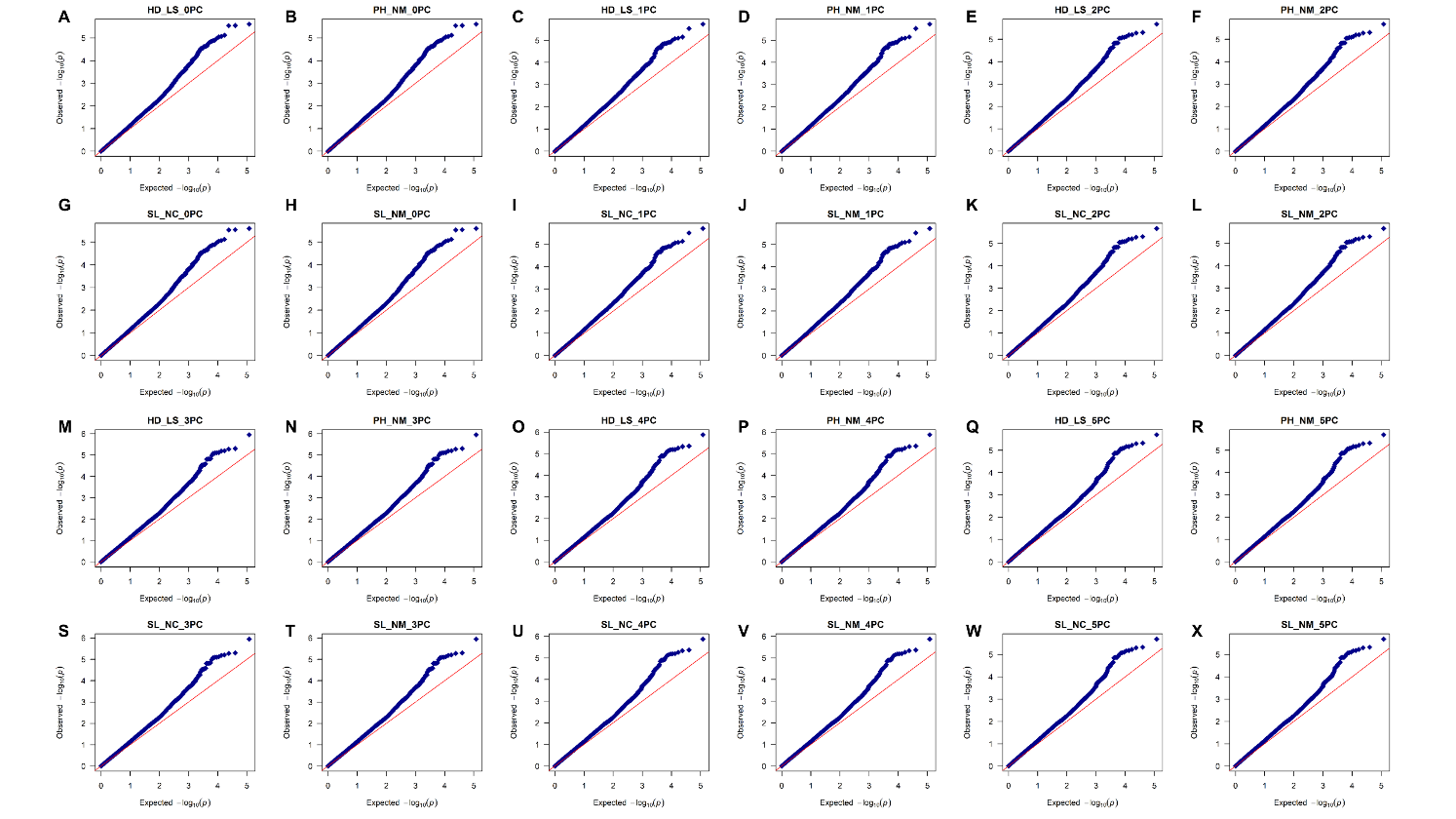


**Supplementary Figure S11.** Quantile-Quantile plots (Q-Q plot) from GWAS. A to X are for heading date in Lhasa (HD_LS), plant height in Namling (PH_NM), spike length in Nyingchi (SL_NC), and spike length in Namling (SL_NM) from MLM with no PC and PC number from 1 to 5 as cofactor, respectively.


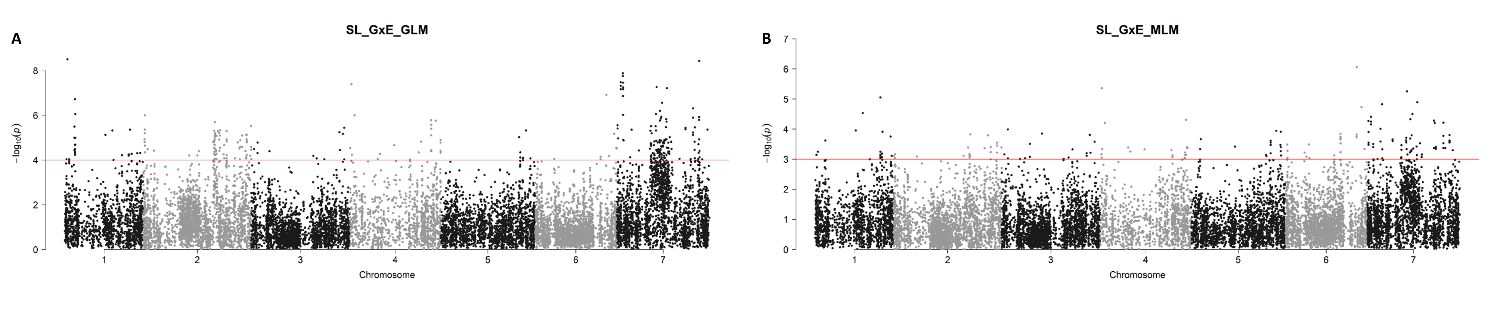


**Supplementary Figure S12.** Manhattan plots of GWAS for spike length across two locations. A and B are the results from GLM and MLM, respectively.


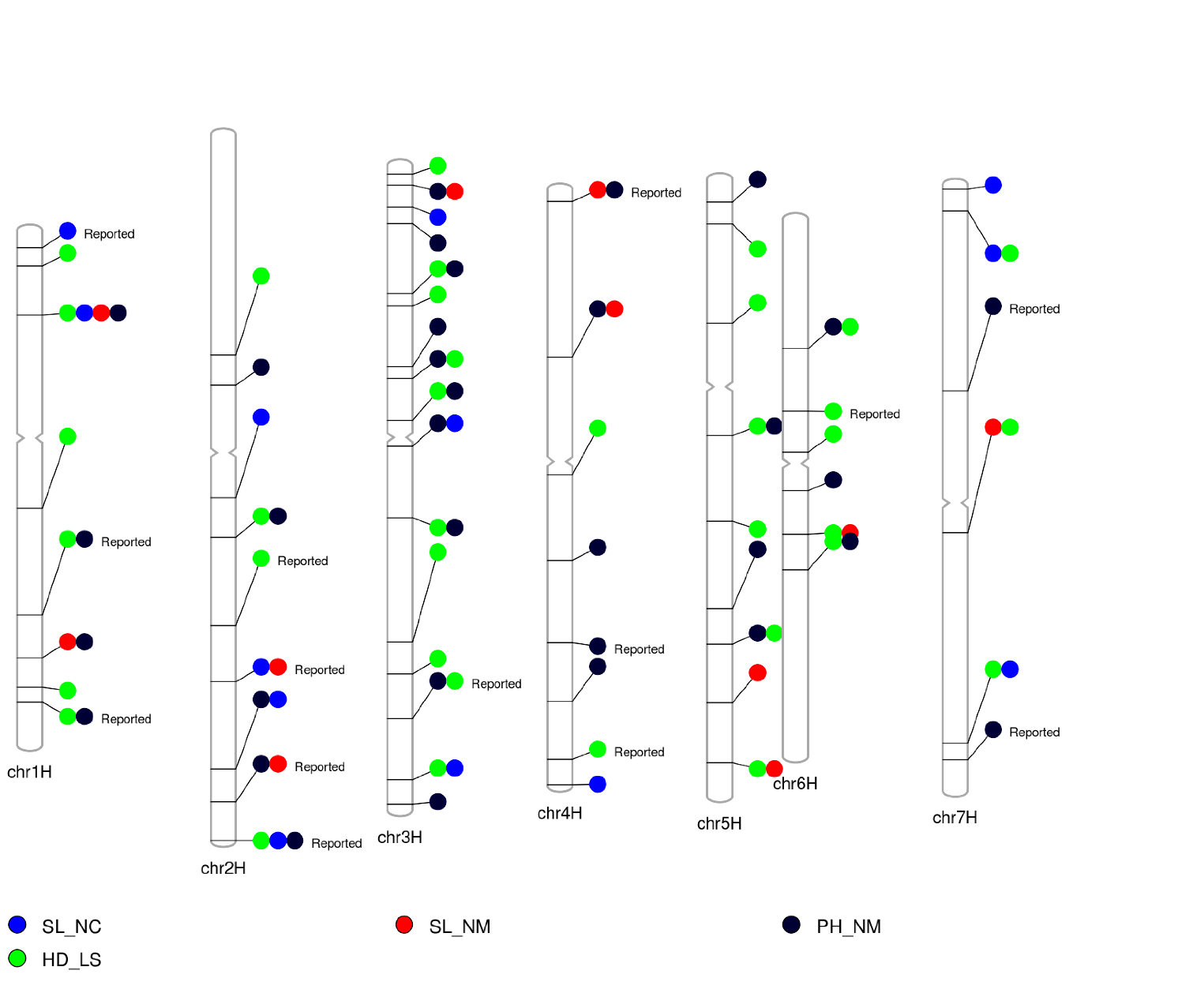


**Supplementary Figure S13.** Distribution of 62 QTLs for heading date in Lhasa (HD_LS), plant height in Namling (PH_NM), spike length in Nyingchi (SL_NC), and spike length in Namling (SL_NM). The QTLs consistent with previous studies are marked by “Reported”.
